# Heliorhodopsin binds and regulates glutamine synthetase activity

**DOI:** 10.1371/journal.pbio.3001817

**Published:** 2022-10-03

**Authors:** Shin-Gyu Cho, Myungchul Song, Kimleng Chuon, Jin-gon Shim, Seanghun Meas, Kwang-Hwan Jung

**Affiliations:** 1 Department of Life Science and Institute of Biological Interfaces, Sogang University, Seoul, Korea; 2 Research Institute for Basic Science, Sogang University, Seoul, Korea; 3 Department of Biology, Faculty of Science, Royal University of Phnom Penh, Phnom Penh, Cambodia; Rutgers University-Robert Wood Johnson Medical School, UNITED STATES

## Abstract

Photoreceptors are light-sensitive proteins found in various organisms that respond to light and relay signals into the cells. Heliorhodopsin, a retinal-binding membrane protein, has been recently discovered, however its function remains unknown. Herein, we investigated the relationship between *Actinobacteria bacterium* IMCC26103 heliorhodopsin (AbHeR) and an adjacent glutamine synthetase (AbGS) in the same operon. We demonstrate that AbHeR binds to AbGS and regulates AbGS activity. More specifically, the dissociation constant (K_d_) value of the binding between AbHeR and AbGS is 6.06 μM. Moreover, the absence of positively charged residues within the intracellular loop of AbHeR impacted K_d_ value as they serve as critical binding sites for AbGS. We also confirm that AbHeR up-regulates the biosynthetic enzyme activity of AbGS both in vitro and in vivo in the presence of light. GS is a key enzyme involved in nitrogen assimilation that catalyzes the conversion of glutamate and ammonia to glutamine. Hence, the interaction between AbHeR and AbGS may be critical for nitrogen assimilation in *Actinobacteria bacterium* IMCC26103 as it survives in low-nutrient environments. Overall, the findings of our study describe, for the first time, to the best of our knowledge, a novel function of heliorhodopsin as a regulatory rhodopsin with the capacity to bind and regulate enzyme activity required for nitrogen assimilation.

## Abstract 2

A study of heliorhodopsin, an actinobacterial photoreceptor of unknown function, reveals that it interacts with glutamine synthetase, an enzyme involved in nitrogen assimilation, and regulates its activity in the presence of light, highlighting the diverse functions of rhodopsins in different organisms.

## Introduction

Organisms from all domains of life use photoreceptor proteins to sense and respond to light. Most rhodopsins, one of the groups of photoreceptor proteins, are light-driven 7-transmembrane retinal-binding proteins found in prokaryotic and eukaryotic organisms. Rhodopsin comprises opsin as apoprotein and retinal as a covalently linked chromophore that absorbs photons for energy conversion or signal transduction [1]. Indeed, rhodopsins play important roles in organism survival. More specifically, type II rhodopsins, which are found mostly in animals, participate in visual and non-visual phototransduction (circadian rhythms, sensing dawn/dark, and determining the horizon) [2–4]. In contrast, type I rhodopsins in microbes participate in the active or passive transport of ions and signal transduction [5].

Light-driven type I rhodopsins have been described as H^+^, Cl^-^, and Na^+^ pumps, light sensors, and channelrhodopsins. They typically contain conserved residues that are specific for the ion that they transport [6–12]. Microbial rhodopsins contain various critical residues, including those within the retinal-binding pocket, retinal covalent linkage, and counterions associated with retinal [13]. N- and C-termini are located on the extracellular and cytoplasmic sides, respectively, in type I rhodopsins. Oligomeric forms of the ion pumps and channelrhodopsins are tri- to penta/hexamers, and dimers, respectively [14–16].

Recently, a new family of microbial rhodopsins, termed heliorhodopsin, has been identified. Heliorhodopsin can be differentiated from type I rhodopsins based on its sequence. That is, the inclusion of heliorhodopsins in the phylogenetic tree of microbial rhodopsins forms distinct clades from type I rhodopsins [17]. Although, heliorhodopsin is also a 7-transmembrane retinal-binding protein, it lacks critical residues involved in pumping ions. Unlike type I and type II rhodopsins, the topology of heliorhodopsin is invertedly embedded; i.e., the N- and C-termini are located on the cytoplasmic and extracellular sides, respectively [17]. Moreover, the oligomeric heliorhodopsin crystal structures revealed a dimer containing several hydrophobic residues, which is not conducive to ion transport [18,19]. In other studies, the binding of divalent cations to heliorhodopsin was studied using attenuated total reflectance-Fourier transform infrared spectroscopy, wherein the binding of Zn^2+^ to heliorhodopsin was detected [20]. Heliorhodopsin photocycle was slow, a feature that is widespread in sensory rhodopsins (SRs). Accordingly, Pushkarev and colleagues suggested that heliorhodopsins function as a distinct type of signaling photoreceptors similar to SRs [17], whereas the N-termini of heliorhodopsins might participate in enzyme function [21].

To further explore the function of heliorhodopsin, we analyzed SRs that interact with other proteins called transducers, which genes are located adjacent to those of SRs, suggesting that a single promoter can transcribe both SR- and transducer-encoding genes [22]. We hypothesized that heliorhodopsin may also interact with other proteins encoded by adjacent genes, similar to SRs. In particular, *Actinobacteria bacterium* IMCC26103 heliorhodopsin (AbHeR) is flanked by glutamine synthetase (GS; AbGS, EC 6.3.1.2) [23]. GS is a central enzyme associated with nitrogen assimilation and is distributed in bacteria as well as plant and animal tissues. GS catalyzes the conversion of glutamate and ammonia to glutamine [24–27]. In fact, GS absence or mutation can lead to reduced growth in bacteria and plants, low grain yield in plants, as well as multi-organ failure and neonatal death in humans [27–29].

Herein, we investigate the relationship between AbGS and AbHeR. Notably, protein–protein interactions (PPIs) were observed between AbHeR and AbGS, which was associated with the regulatory function of AbHeR for AbGS. Here, we report, for the first time, to the best of our knowledge, one of the functional roles for AbHeR in regulating AbGS activity.

## Results

### AbHeR interacts with AbGS

**Fig 1:**
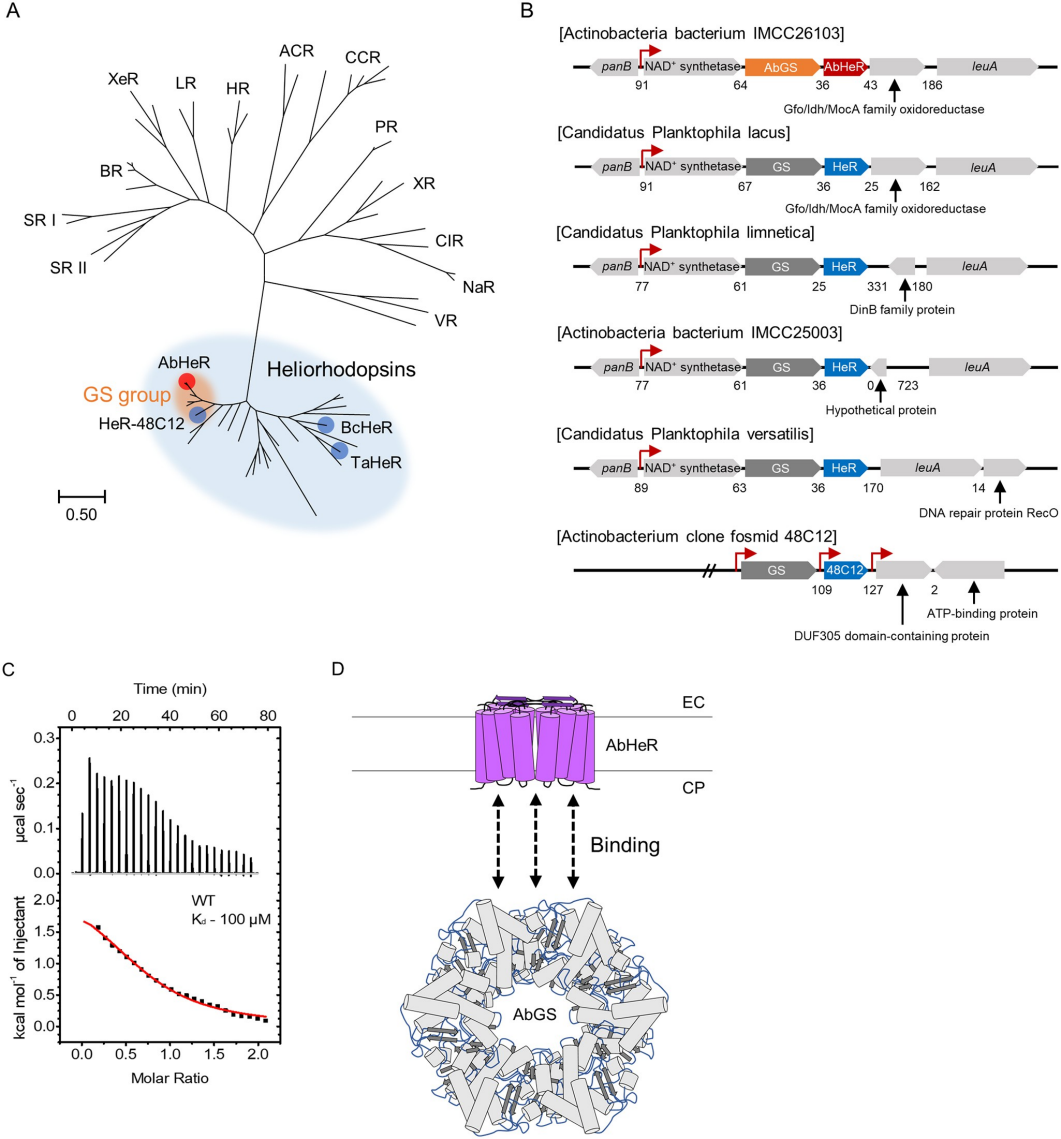
Identification of AbGS binding to AbHeR. (A) Relationship between microbial rhodopsin and heliorhodopsin. The unrooted maximum likelihood tree is shown. Blue oval, blue circle, red circle, and orange oval represent heliorhodopsins clade, reported heliorhodopsins, AbHeR, and heliorhodopsins containing GS gene, respectively. (B) Each eubacterium contains GS and heliorhodopsin arranged in operons in the indicated direction. Nucleotide gaps between HeR and GS are indicated. AbGS and AbHeR are indicated by orange and red arrows, respectively. Predicted GS and other heliorhodopsin are indicated by gray and blue arrows, respectively. Promoters in each operon are expressed by red bent arrows and predicted using phiSITE, Softberry, BDGP, and SAPPHIRE. (C) PPI between AbHeR and AbGS is determined by isothermal titration calorimetry analysis. The upper and lower panels represent raw data and enthalpy change per mol, respectively. AbGS was continuously added to AbHeR wild type. Nonlinear curves in the lower panels evaluated the best-fit curve. (D) Predicted model of PPI between AbHeR and AbGS. AbHeR dimers are embedded in the membrane, and the N- and C-termini of AbHeR are located in the CP side and EC side, respectively. AbGS dodecamer binds to AbHeR dimers and the potential of interactions between them is indicated by dotted black arrows. The underlying data of the graph can be found in S1 Data. ACR, anion channelrhodopsin; BR, bacteriorhodopsin; CCR, cation channelrhodopsin; CIR, Cl^–^-pumping rhodopsin; CP, cytoplasmic; EC, extracellular; GS, glutamine synthetase; HR, halorhodopsin; LR, *Leptosphaeria* rhodopsin; NaR, Na^+^-pumping rhodopsin; PPI, protein–protein interaction; PR, proteorhodopsin; SR I, sensory rhodopsin I; SR II, sensory rhodopsin II; VR, viral rhodopsin; XeR, xenorhodopsin; XR, xanthorhodopsin.

The N-terminus of HeR-48C12, the first heliorhodopsin to be studied, faces the cytoplasmic side, whereas in type I rhodopsins, the C-terminus faces the extracellular side [17–19]. AbHeR in *Actinobacteria bacterium* IMCC26103 exhibited sequence similarity with HeR-48C12, a novel class of heliorhodopsin, which is not the type I rhodopsin (Fig 1A). In addition, the N-terminus of AbHeR was located on the cytoplasmic side, similar to previously known heliorhodopsins, as predicted by the Philius server [30] (S1A Fig), and based on the crystal structures [18,19,31].

The retinal-binding pocket and functional key motifs of type I rhodopsins comprise relatively conserved amino acid sequences [13]. However, we found that the functional key motif in bacteriorhodopsin (BR), Asp85, Thr89, and Asp96, were replaced with Glu105, Ser109, and Leu116, respectively, in AbHeR, suggesting that AbHeR has functions that differ from those of previously characterized type I rhodopsins (S2A Fig). Regarding the counterion positions, Asp212 in BR was replaced with Ser235 in AbHeR [13]. The retinal-binding pocket in AbHeR also contained aromatic amino acids (Tyr106, Phe204, and Phe207) similar to BR (Trp86, Trp182, and Tyr185); however, Trp189 in BR was replaced with Gln211 in AbHeR, suggesting that the positions of the retinal-binding pocket were fairly preserved. In addition, covalent linkage with retinal (Lys216 in BR) was conserved in AbHeR (S2A Fig) [13]. We then compared AbHeR with other heliorhodopsins and found that the functional key motif, counterion, retinal-binding pocket, and retinal covalent linkage in AbHeR were highly conserved (S2B Fig). Therefore, AbHeR appears to belong to the class of heliorhodopsins [19].

Kovalev and colleagues analyzed structure-based bioinformatics of heliorhodopsins with HeR-48C12 and classified the heliorhodopsins into 10 subfamilies based on the amino acid sequences [19]. By applying their classification system, AbHeR was observed to be similar to HeR-48C12 (subfamily 1) as they exhibited a high degree of amino acid sequence similarity within the hydrophobic barrier, retinal Schiff base, cavity, the hydrophobic region, as well as the region near Glu149 of HeR-48C12. Most conservative residues in subfamily 1 differ from those of other subfamilies. For instance, many subfamily 2 members contained Glu in the Ile200 position in AbHeR (Leu202 in HeR-48C12), which belongs to its hydrophobic extracellular region. Additionally, Ala166 and Pro170 in AbHeR (Ala168 and Pro172 in HeR-48C12) were replaced by Pro and other amino acids in subfamily 2, respectively. Meanwhile, in subfamilies 3 to 5, Asn and Met were placed in the Ile140 and Gln211 in AbHeR (Ile142 and Gln213 in HeR-48C12), respectively. Furthermore, subfamilies 7 to 9 contain a conserved Tyr in the Ile200 in AbHeR (Leu202 in HeR-48C12). Finally, the subfamily of unsorted proteins group was the most different when compared with AbHeR (S2B Fig). Hence, collectively the comparisons revealed that AbHeR belongs to subfamily 1.

Subsequently, we determined the photochemical and biophysical properties of AbHeR. The absorption maxima, as well as estimated p*K*a values of Glu105 and retinal Schiff base were 553 nm, 2.84 (± 1.07), and 11.72 (± 2.05), respectively (S1B–S1F Fig). To probe the ion-pumping activity, light-induced right-side-out (RSO) membrane vesicles containing AbHeR were analyzed with 100 mM NaCl and a mixture solution comprising 20 mM each of LiCl, NaCl, KCl, CsCl, and Na_2_SO_4_; however, AbHeR did not pump any ions with or without carbonyl cyanide *m*-chlorophenyl hydrazone (CCCP; S1G Fig). The results suggest that AbHeR is not a light-driven ion pump.

Microbial rhodopsins exhibit signal transduction as well as ion pumping in the presence of light. Microbial SRs assist bacteria in swimming toward favorable light or away from unfavorable light by interacting with the transducer. In addition, SRs and transducers are regulated by a single promoter within the genome [22]. We hypothesized that heliorhodopsins may interact with the proteins by genes up- or downstream of the heliorhodopsins and function as a signal transduction protein or regulator. To probe this hypothesis, we analyzed the AbHeR operon and observed *panB*, and nicotinamide adenine dinucleotide (NAD^+^) synthetase, AbGS, Gfo/Idh/MocA family oxidoreductase, and *leuA* genes (Fig 1B). A promoter in the operon was predicted upstream of the NAD^+^ synthetase gene, which may transcribe 4 genes (NAD^+^ synthetase, AbGS, AbHeR, and Gfo/Idh/MocA family oxidoreductase genes). In addition, terminators in the nucleotide gaps were not predicted from NAD^+^ synthetase to Gfo/Idh/MocA family oxidoreductase genes. That is, the genes may be under the control of a single promoter. We assumed that there may be more bacteria with similar operons, including heliorhodopsin. Furthermore, excluding the *Actinobacterium* clone fosmid 48C12 encoding HeR-48C12 gene, operons of other bacteria were also regulated by a single promoter (Fig 1B). Since various genes are regulated by the single promoter, we focused on the adjacent gene, AbGS, which was upstream of AbHeR. Interestingly, heliorhodopsins with adjacent GS genes were located in proximity to each other in the phylogenetic tree and were grouped into the GS group. In fact, the degree of amino acid similarity between heliorhodopsins in the GS group was 74% to 92% (Fig 1A).

Understanding the biophysical principles of PPIs is essential for the comprehension of their physiological role in cells [32]. The biochemical events associated with PPIs include electrostatic forces, hydrogen bonding, and hydrophobic effects, making them important factors in various biosystems [32,33]. PPIs between microbial rhodopsin and transducers have been reported, and their thermodynamic parameters have been characterized using isothermal titration calorimetry (ITC), which is an analytical method used to measure thermal changes with high sensitivity [33]. Purified AbHeR wild type (WT) and AbGS were quantified, and AbGS was continuously injected into AbHeR using the ITC injector at 25°C. The dissociation constant (K_d_), enthalpy (ΔH), and Gibbs free energy (ΔG) values for the binding of AbHeR to AbGS in a detergent (n-dodecyl-β-D-maltopyranoside, DDM), which might affect the binding between AbGS and AbHeR, were determined to be 100 (±20) μM, 2,072 (±238.6) cal mol^-1^, and −5,411 cal mol^-1^, respectively (Fig 1C and Table 1). The binding between AbHeR and AbGS is interesting as AbGS, a soluble protein, has limited sites that can bind to membrane protein (Fig 1D). Therefore, were probed the binding site between AbHeR and AbGS, to determine if the binding alters the activity of AbGS.

**Table 1:** Binding affinities of AbGS for AbHeR WT and mutants.

AbHeR	K_d_ (μM)	ΔH	ΔS	ΔG
WT	100 (±20)	2,072 (±238.6)	25.1	−5,411
E147Q	NE			
K148Q	137 (±44)	5,308 (±775.0)	35.5	−5,276
E150Q	NS			
E151Q	30 (±8)	2,358 (±247.2)	28.4	−6,110
K216Q^a^	3,676 (±5,595)	4.401E7 (±1.032E8)	1.48E5	−116,200
K217Q^a^	537 (±1,751)	9,757 (±2.283E4)	47.7	−4,465
K220Q^a^	833 (±2,840)	9,366 (±2.45E4)	45.5	−4,200
D223N	44 (±23)	1,135 (±188.7)	23.7	−5,931
E228Q	NE			

^a^ Indicates no binding.

NE, not expressed; NS, not solubilized; WT, wild type.

### Binding affinity of AbGS for mutated AbHeR

**Fig 2:**
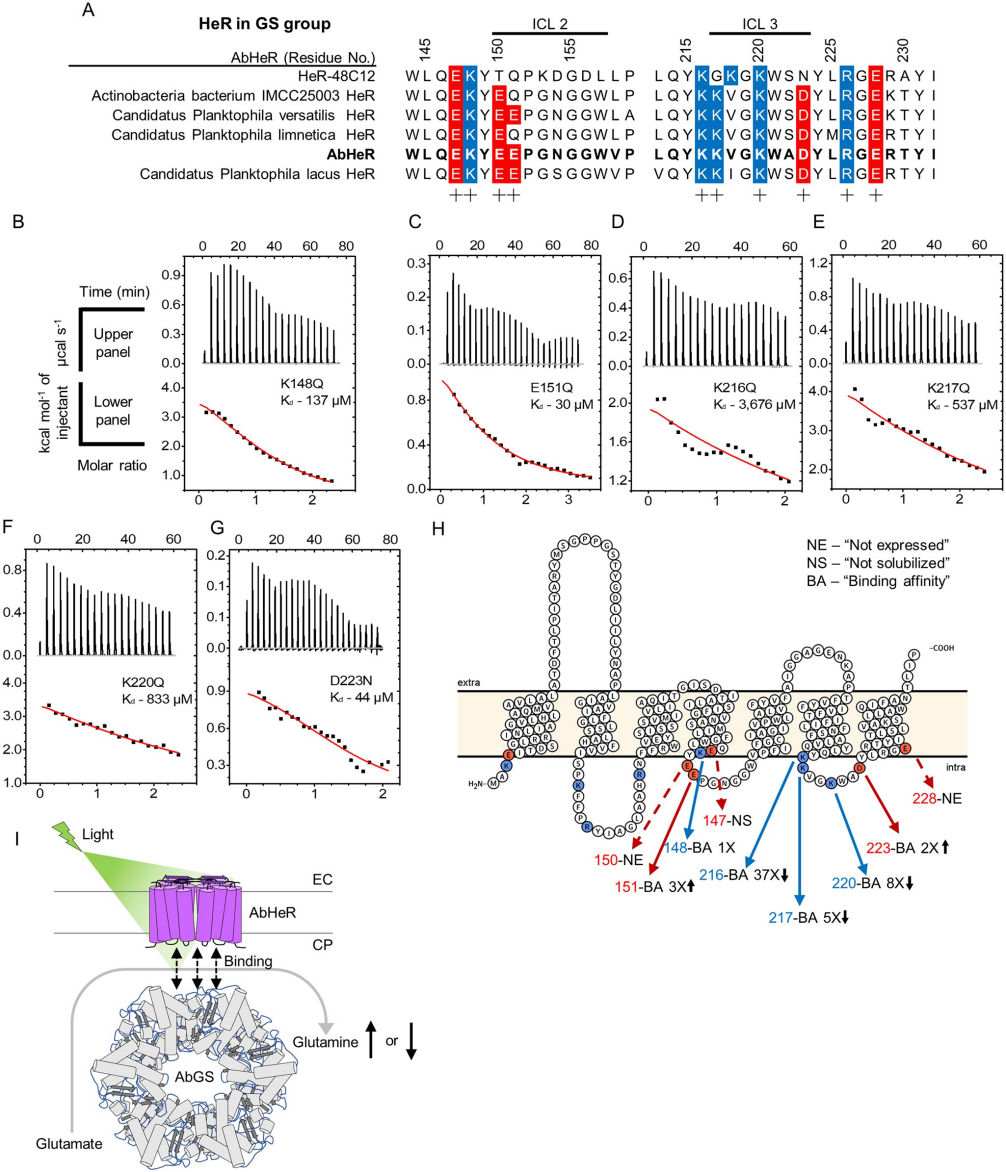
PPIs and binding affinities of AbHeR for AbGS. (A) Multiple sequence alignments of AbHeR and predicted heliorhodopsin in the GS group were aligned using MUSCLE. Alignments of AbHeR with heliorhodopsin in the GS group. Negatively and positively charged residues near intracellular loops of heliorhodopsin are indicated by red and blue, respectively. (B–G) Dissociation constants of AbHeR for AbGS determined using ITC analysis. The upper and lower panels represent raw data and enthalpy change per mol, respectively. AbGS was continuously added to AbHeR mutants. Nonlinear curves of lower panels were evaluated by the best-fit curve. (H) Scheme of PPIs in each amino acid position of AbHeR for AbGS, as predicted by Protter. Positively and negatively charged residues on the cytoplasmic side are indicated as red and blue circles, respectively, with arrows. Dotted arrows indicate not expressed and solubilized proteins. Fold-changes for AbHeR mutants compared to WT indicate text with black arrows. (I) Predicted model of AbHeR function associated with AbGS. AbHeR dimers are embedded in the membrane, and the N- and C-termini of AbHeR are located on the CP and EC sides, respectively. AbGS dodecamer binds to AbHeR dimers and exhibits altered enzyme activity in the presence of light. The underlying data of the ITC analysis can be found in S1 Data. CP, cytoplasmic; EC, extracellular; GS, glutamine synthetase; ITC, isothermal titration calorimetry; MUSCLE, MUltiple Sequence Comparison by Log-Expectation; PPI, protein–protein interaction; WT, wild type.

Next, we determined the AbHeR residues involved in AbGS binding. Considering AbGS is a soluble protein, we postulated that the binding residues in AbHeR were located on the cytoplasmic side. We identified AbHeR charged residues on the intracellular loop (ICL) using Protter [34] and amino acid alignment tools (Fig 2A and 2H). AbHeR and AbGS were predicted to be in dimer and dodecamer forms, respectively (S3A–S3D Fig). To elucidate the role of electrostatic forces in the associated PPIs, we used an electrostatic potential map and found that AbGS did not form a field with a dominant charge in a specific area (S3E Fig). Consequently, we analyzed the electrostatic potential map for AbHeR (S3F Fig). Notably, AbHeR exhibited negatively and positively charged areas on the cytoplasmic side. The positively charged area included K3, K217, K216, K220, and R226, in the front and side membrane views. In the bottom cytoplasmic side view, the negatively charged area included E150, E151, and D223. Overall, there were more positively charged areas than negatively charged fields; K3, K216, K217, K220, and R226 formed the field in the front-side-bottom view (cytoplasmic to extracellular side; S3F Fig).

We confirmed that the charged residues of heliorhodopsins on ICL2 and ICL3 were conserved compared to in the ICL1 region and N-terminus (S2B Fig). In particular, charged residues of heliorhodopsins in the GS group were highly conserved on the ICL2 and ICL3 (Fig 2A). Hence, we constructed the following AbHeR mutants with alterations in ICL2 and ICL3 regions: E147Q, K148Q, E150Q, E151Q, K216Q, K217Q, K220Q, D223N, and E228Q. The AbHeR E147Q mutant was successfully expressed, however, was not solubilized in 1% DDM. AbHeR E150Q and E228Q mutants were not expressed. The AbHeR K148Q, E151Q, K216Q, K217Q, K220Q, and D223N mutants were successfully expressed and purified, with the absorbance maxima remaining unaltered (S1H Fig).

We performed ITC analysis for the AbHeR mutants with AbGS. The K_d_ values of K148Q and E151Q on ICL2 was determined to be 137 (±44) and 30 (±8) μM, respectively (Fig 2B and 2C, and Table 1). The K_d_ value of K148Q was similar to that of WT, indicating that the K148 position was not influenced by AbGS binding. However, the K_d_ value of E151Q decreased by approximately 3 times (Fig 2H and Table 1). The K_d_ values of K216Q, K217Q, K220Q, and D223N on ICL3 were determined to be 3,676 (±5,595), 537 (±1,751), 833 (±2,840), and 44 (±23) μM, respectively (Fig 2D–2G and Table 1). Three positively charged residues on ICL3 influenced the binding to AbGS, and the K216 position exhibited extremely weak binding when compared with WT, resulting in an approximate 37-fold increase in the K_d_ value of K216Q (Fig 2H and Table 1). Hence, we propose that the K216 position is critical for binding of AbGS and that the K217 and K220 residues facilitate the binding. The K_d_ value of D223N also decreased by approximately 2 times similar to E151Q, suggesting that the absence of negatively charged residues is required for AbGS binding (Fig 2H and Table 1). SRs can respond to various light stimuli and regulate the transducer for signal transduction in different organisms [11]. Similarly, the PPI between AbHeR and AbGS may regulate AbGS enzyme activity in the presence of light, which may influence the growth or light stimuli of *Actinobacteria bacterium* IMCC26103 (Fig 2I).

### AbHeR regulates AbGS activity

GS is one of the key enzymes that participate in nitrogen metabolism, as it catalyzes glutamine synthesis from glutamate via the following reaction (Eq 1):

$$\text{Glutamate}+{\text{NH}}_{4}{}^{+}+{\text{ATP}}^{\text{Me}2+}\;\text{glutamine}+\text{ADP}+{\text{P}}_{i}+{\text{H}}^{+}.$$ (1)

The biosynthetic reactions include conversion of L-glutamate, in the presence of ATP, ammonia, and metal ion (Me^2+^, magnesium, or manganese), to L-glutamine with adenosine diphosphate (ADP), inorganic phosphate (P_*i*_), and a proton as by-products [35]. We performed a biosynthesis assay rather than the γ-glutamyltransferase assay as the procedures of the latter involve the use of hydroxylamine, which affects rhodopsin bleaching [36]. The biosynthesis assay involves measuring the concentration of P_*i*_ after GS catalysis and colorimetric determination of P_*i*_ with ferric sulfate and molybdate [37,38]. AbGS activity was calculated using a standard curve for P_*i*_ concentration (S4A Fig), and enzyme kinetic parameters of AbGS were determined (Table 2 and S4B and S4C Fig). *k*_cat_ and *k*_cat_/*K*_m_ values of reported recombinant GSs for L-glutamate were 2.0 to 30.5 s^-1^ and 900 to 28,000 M^-1^s^-1^, respectively [39–41], implying that AbGS activity is lower than those of the reported recombinant GSs.

**Table 2:** Steady-state enzyme kinetic parameters for AbGS without and with AbHeR in the absence and presence of light in a Mn^2+^-supported biosynthetic reaction.

AbGS and AbHeR	*K*_m_^a^ (mM, Glu)	*V*_max_^a^ (μM/min)	*k*_cat_ (s^-1^)	*k*_cat_/*K*_m_ (M^-1^s^-1^)
GS	27.05 (±1.65)	140.76 (±2.13)	0.1466 (±0.022)	5.42
GS + WT (Dark)	12.75 (±2.12)	98.02 (±2.94)	0.1021 (±0.025)	8.01
GS + WT (Light)	9.46 (±0.70)	110.65 (±1.21)	0.1153 (±0.013)	12.19
GS + K216Q (Dark)	18.72 (±7.53)	97.96 (±8.92)	0.1020 (±0.093)	5.45
GS + K216Q (Light)	14.53 (±5.51)	96.43 (±7.15)	0.1004 (±0.082)	6.91

^a^ *K*_m_ and *V*_max_ were calculated using the Michaelis–Menten equation.

Values are mean ± standard deviation (*n* = 3).

The biosynthetic reactions were carried out at 37°C.

Next, to stabilize AbHeR for heat and illumination, we prepared an inverted membrane vesicle (IMV), (inside-out (ISO) membrane vesicle) with an orientation opposite that of RSO membrane vesicles [42,43]. We predicted that the exposed cytoplasmic side of AbHeR IMV would readily bind AbGS. The absorption spectra of the prepared AbHeR IMV and empty IMV were measured; both AbHeR WT and K216Q IMV exhibited absorption maxima similar to that of purified AbHeR (S1B and S1H and S5A Figs). To check the reverse direction of IMV, a light-induced H^+^ movement assay was performed with *Gloeobacter* rhodopsin (GR) WT as a light-induced H^+^-pumping rhodopsin [44]. The GR WT IMV pumped H^+^ inward, compared with GR WT (S5B Fig), suggesting that our prepared IMV was successfully inverted. Moreover, AbHeR IMV exhibited thermal and light stability (S5C Fig).

**Fig 3:**
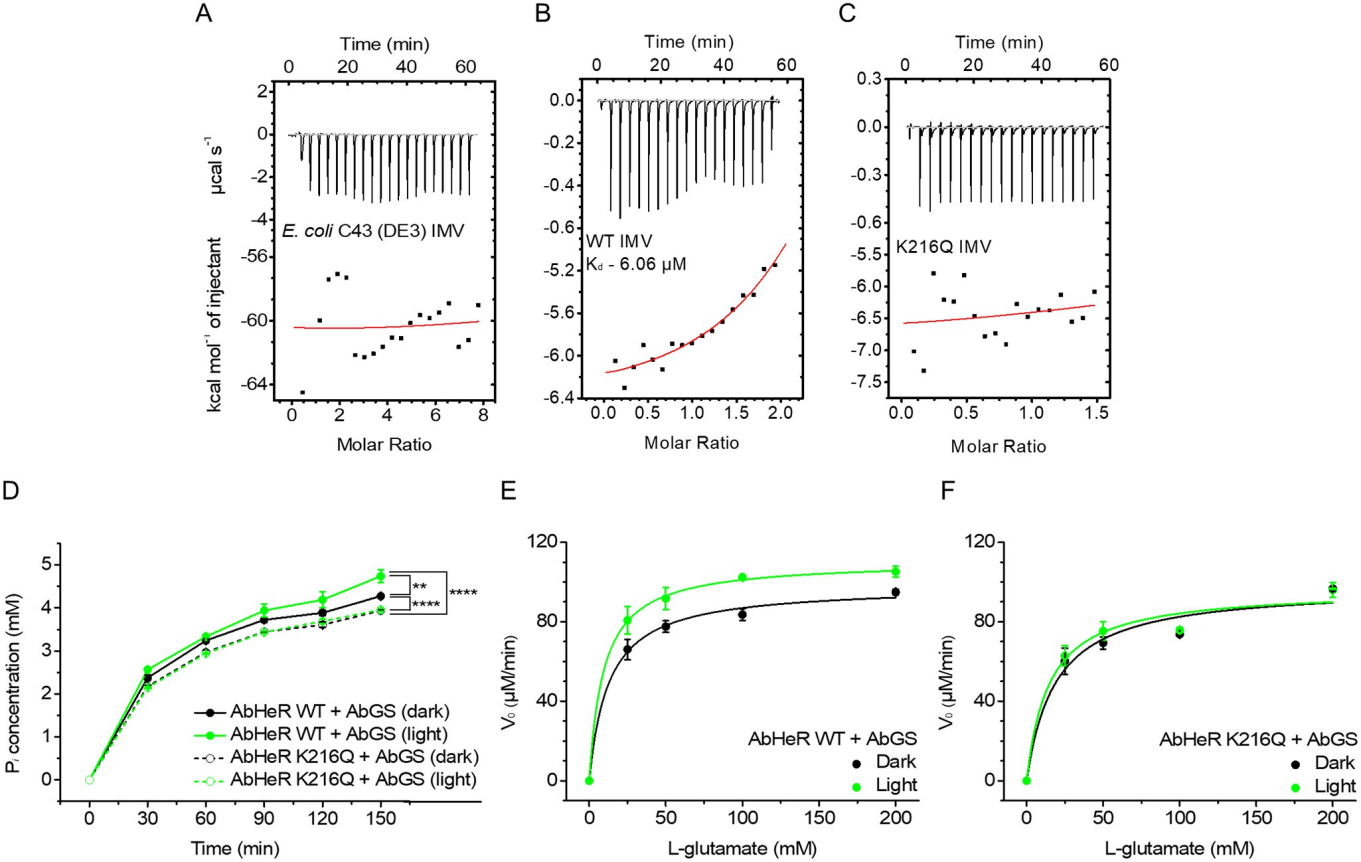
AbGS activity is regulated in vitro via AbHeR binding. The binding of AbGS to preparations of *Escherichia coli* C43 (DE3) (A), AbHeR WT (B), and AbHeR K216Q (C) IMVs were analyzed by ITC analysis. The upper and lower panels represent the raw data and enthalpy change per mol, respectively. AbGS was continuously added to IMVs. The nonlinear curves of the lower panels were evaluated by best-fit curve analysis. (D–F) AbGS reactions were performed with AbHeR WT and K216Q IMV in the absence (dark) and presence of light (light, 532 nm) for time traces at 30 μmol m^-2^ s^-1^ using a biosynthesis assay. The nonlinear and linear fits were evaluated using the Michaelis–Menten equation (E and F). (D) Biosynthesis assay of AbGS reactions with AbHeR WT IMV carried out with 100 mM L-glutamate at different times. The measurements were conducted in an independent experimental group (*n* = 6), and the data are expressed as mean ± standard deviation. The statistical significance between the 2 groups was analyzed using the *t* test; *p*-values are indicated by an asterisk. The underlying data of the ITC analysis and the biosynthesis assay can be found in S1 and S2 Data, respectively. IMV, inverted membrane vesicle; ITC, isothermal titration calorimetry; WT, wild type.

As AbHeR WT binds to AbGS, we postulated that AbHeR WT may influence AbGS activity. To test this hypothesis, we employed the AbHeR K216Q mutant as a control, which was not expected to influence AbGS activity due to its lack of binding activity. To confirm PPIs between AbHeR IMV and AbGS, the associated binding affinity was analyzed using ITC analysis. The IMVs were continuously injected into the AbGS, wherein *Escherichia coli* C43 (DE3) and AbHeR K216Q IMVs were not observed to be bound (Fig 3A and 3C). However, AbHeR WT IMV was bound to AbGS. These results are consistent with the ITC data of purified AbHeR. The K_d_ value of AbHeR WT IMV was determined to be 6.06 (± 5.44; Fig 3B). Interestingly, the binding affinity of AbHeR IMV for AbGS (Fig 3B) was improved when compared with that for purified AbHeR with AbGS (Fig 1C). The only difference between the proteins was the absence or presence of detergent (DDM). DDM stabilizes membrane proteins; however, it may bind to several areas of AbGS as a soluble protein. Therefore, areas of AbGS do not interfere with the binding to AbHeR. Consequently, we suggest that AbGS suitably binds to AbHeR in the *E*. *coli* membrane and can be used for subsequent in vivo experiments. Furthermore, AbHeR may bind to AbGS in living cells.

AbHeR IMV, is heat and light stable, binds to AbGS, and is suitable for studying the effect of AbHeR on AbGS. We designed a biosynthesis assay to investigate the effect of AbHeR in the presence of light, wherein the reactions of AbHeR IMV with AbGS were subtracted from AbHeR IMV alone because ATP hydrolysis by embedded membrane proteins in IMV can be influenced. The AbGS activity with AbHeR WT was statistically significantly increased in the presence of light, implying that AbGS was regulated by light-induced AbHeR (Fig 3D). Consistent with the ITC analysis results, AbHeR K216Q did not bind to AbGS and was not significantly altered in the presence of light (Fig 3D). In the absence of light, the amount of a product (P_*i*_) produced by AbGS bound to AbHeR WT at 150 min was increased 1.09 times when compared with that in AbHeR K216Q. In the presence of light, the amount of the product produced by AbGS bound AbHeR WT at 150 min was increased 1.1 times when compared with that observed in the absence of light. When compared with AbGS with AbHeR K216Q, the amount of the product produced by AbGS bound to AbHeR WT at 150 min was increased 1.2 times in the presence of light. Therefore, AbGS is more productive when bound to AbHeR than in its unbound state; the effect is stronger in the presence of light.

For more accurate comparisons of AbGS activity, we quantified constant values through the enzyme kinetic parameters of AbGS with AbHeR in the absence and presence of light (Table 2 and Fig 3E and 3F). In the absence of light, *K*_m_ and *k*_*cat*_ values of AbGS with AbHeR WT were lower than those for AbGS alone. In the presence of light, the *K*_m_ and *k*_*cat*_ values were lower and greater, respectively, suggesting increased activity. Although the activity of AbGS with AbHeR WT was greater in the presence of light, the *K*_m_ and *k*_cat_ values were lower under such conditions than under AbGS alone. However, as the values differ for each condition, comparing the actual AbGS activity is challenging. It is more appropriate to compare the catalytic efficiencies based on the assessment of the specificity constant (i.e., *k*_cat_/*K*_m_ ratio). In the absence of light, the *k*_cat_/*K*_m_ value of AbGS in the presence of AbHeR WT was 1.52 times greater than that for AbGS alone. In the presence of light, the *k*_cat_/*K*_m_ value of AbGS in presence of AbHeR WT was 2.24 times greater than that for AbGS alone. Moreover, the AbHeR K216Q, mutation of a critical binding residue for AbGS, did not regulate AbGS significantly when compared with AbGS alone; however, the *k*_cat_/*K*_m_ value of AbGS in the presence of AbHeR K216Q and light was 1.27 times greater than that in the absence of light. According to the ITC results, there was weakly binding between this mutant and AbGS, resulting in an increase its activity in the presence of light. In a nonessential activation model, which is similar to reversible mixed inhibition, enzyme activity is activated, resulting in changes in both *K*_m_ and *V*_max_ [45]. Therefore, AbHeR might function as a nonessential activator for AbGS.

Since the ITC analysis is performed in the absence of light, AbGS can bind to AbHeR in the absence of light. That is, AbGS binds to the interaction residues (K216 or K216 with other residues) of AbHeR on the cytoplasmic side, followed by conversion of all-*trans* retinal (ATR) to 13-*cis* retinal in AbHeR, resulting in a conformational change in AbHeR in the presence of light. In contrast, AbGS cannot bind AbHeR in the absence of the positively charged residue (K216). Therefore, we suggest that conformational changes in AbHeR influence the interaction with, and activity of, AbGS, and that AbHeR up-regulates AbGS activity.

### Growth rate test of cells with AbGS and AbHeR

We hypothesized that co-expression of AbHeR and AbGS in a cell would up-regulate AbGS activity in the presence of light. *E*. *coli* K-12 MG1655 and JW3841 strains (*glnA* knockout (KO) strain derived from K-12 MG1655) were used as the positive control and experimental group to study the influence of the absence of *glnA* encoding GS alone, respectively. Cellular growth rate was assessed in *E*. *coli* K-12 MG1655 and JW3841 strains to compare the complementation of AbGS with AbHeR.

We added L-glutamate to 3-morpholinopropane-1-sulfonic acid (MOPS) minimal medium to induce AbGS activity. The K-12 MG1655 strain was transformed with the all-in-one (AIO) plasmid containing ampicillin and kanamycin as the *glnA* KO strain exhibited kanamycin resistance. The *glnA* KO strain was transformed with pKA001 vector containing AbGS and AbHeR [46], which were regulated by P_*lac*_ and P_*araBAD*_, respectively. The growth rate of cells was then measured in the absence or presence of L-glutamate, which is required for the catalytic activity of AbGS. Finally, we assessed the growth rate of cells upon complementation of AbGS with AbHeR.

**Fig 4:**
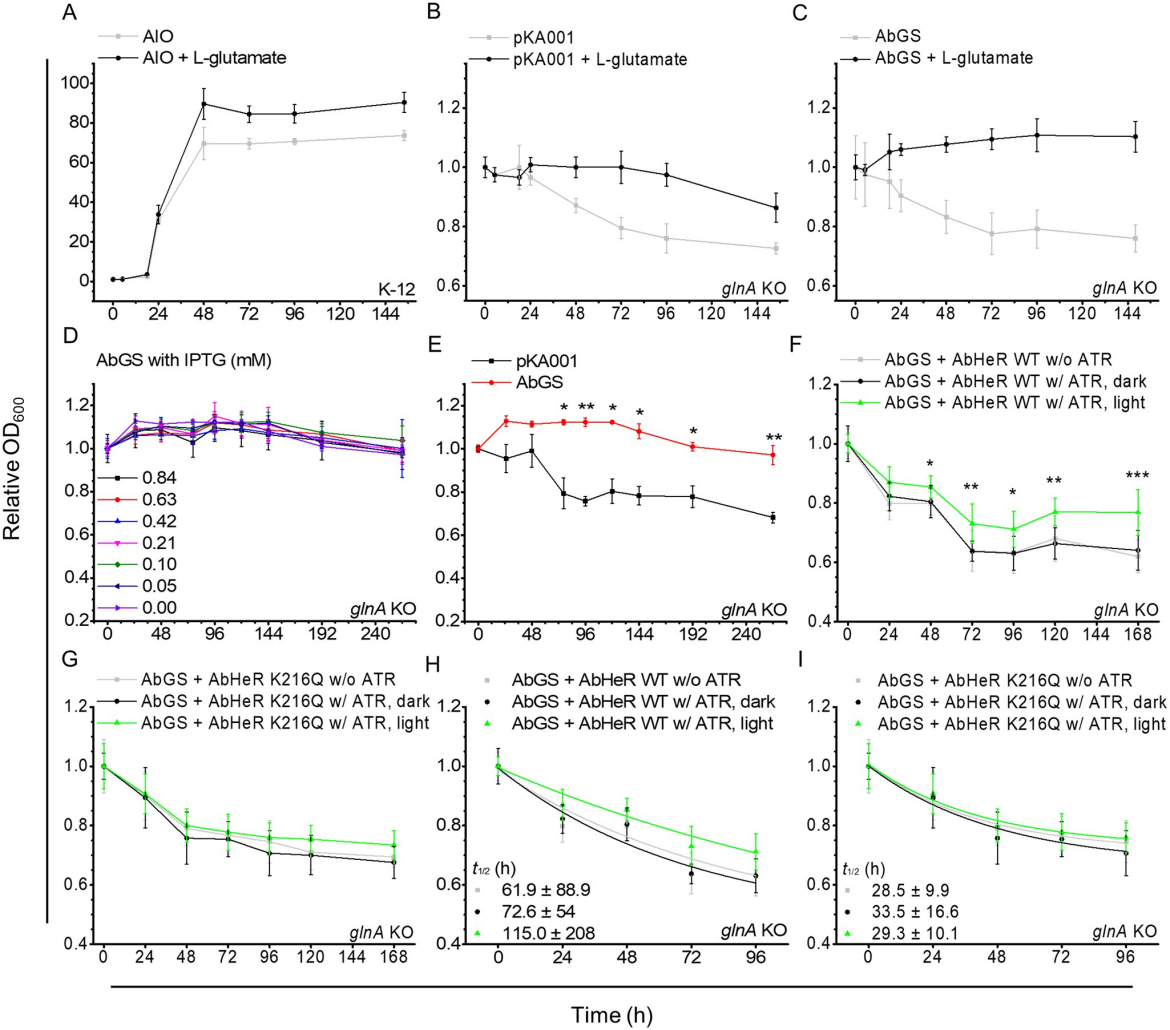
In vivo growth rate of *E*. *coli glnA* KO strains with AbGS and AbHeR. Cells are grown in LB medium and washed to remove components of the LB medium. The cells are then transferred to MOPS minimal medium. The growth of transferred cells was recorded at OD_600_ at different time points; growth is normalized to the initial recording. The *E*. *coli* K-12 MG1655 strain transformed with AIO vector (A), *glnA* KO strain transformed with pKA001 null vector (B), and *glnA* KO strain transformed with pKA001-GS vector (C) were incubated in the absence and presence of L-glutamate in MOPS minimal medium containing IPTG, which regulates AbGS expression. (D–G) The *E*. *coli glnA* KO strain transformed with pKA001 null or pKA001-GS vector was incubated in MOPS minimal medium containing L-glutamate and glycerol (MOPS-GG). The *E*. *coli glnA* KO strain transformed with pKA001-GS-AbHeR WT (F) and K216Q (G) with and without ATR in the absence (dark) and presence of light (light, 532 nm) conditions at 40 μmol m^-2^ s^-1^ were incubated in MOPS-GG medium. (A–E) The tests were performed in an independent experimental group (*n* = 3). (F and G) The tests were performed in an independent experimental group (*n* = 9). (E) Statistical significance between the 2 groups (pKA001 and AbGS) was analyzed using the *t* test; *p*-values are indicated with asterisks. (F and G) Statistical significance between the 2 groups (dark and light) was analyzed using the *t* test; *p*-values are indicated with asterisks. The data are expressed as mean ± standard deviation. Based on data (F) and (G), half-life (*t*_1/2_) for growth rate of cells was analyzed to quantify the effect of AbGS binding in the absence and presence of light by exponential decay. Nonlinear fitted lines are shown for AbHeR WT (H) and K216Q (I). Quantified *t*_1/2_ values are indicated by the same color as that of each rectangle, circle, and triangle. The underlying data of the graph can be found in S3 Data. AIO, all-in-one; KO, knockout; LB, Luria–Bertani; WT, wild type.

The K-12 MG1655 strain was grown in the absence of L-glutamate and saturated for 48 h. In the presence of L-glutamate, the cells were saturated at a higher cell density, indicating that supplementary L-glutamate influenced cell density and growth rate (Fig 4A); i.e., the MOPS minimal medium in place of Luria–Bertani (LB) medium is sufficient to permit cell growth. The *glnA* gene in *E*. *coli* is the key enzyme involved in nitrogen fixation; *glnA*-deficient *E*. *coli* strain reportedly does not grow in minimal mediums [47–49]. Therefore, we postulated that if AbGS catalyzes L-glutamate to L-glutamine in the *glnA* KO strain in the presence of L-glutamate, it would exhibit cell growth similar to that of the K-12 MG1655 strain. As expected, the *glnA* KO strain containing the null vector (*glnA* KO + pKA001) did not grow in MOPS minimal medium. Upon the addition of L-glutamate, cell density began to decrease after 72 h (Fig 4B). Similarly, the *glnA* KO strain expressing AbGS (*glnA* KO + AbGS) did not grow in the absence of L-glutamate. Meanwhile, the cells showed slow growth and became saturated at 72 h in the presence of L-glutamate, indicating that AbGS successfully converted L-glutamate to L-glutamine (Fig 4C). However, the complementation was not recovered to the level exhibited by the K-12 MG1655 strain, suggesting that AbGS activity is lower than that of the well-known GS.

L-glucose, as a carbon source in the MOPS minimal medium, can interfere with isopropyl β-D-1-thiogalactopyranoside (IPTG), which regulates AbGS expression on the *lac* operon; therefore, we used glycerol as the carbon source in the MOPS minimal medium. In addition, we assumed that the medium supplemented with glycerol provides a limited source of energy for growth. The synthesis of recombinant proteins expressed by IPTG may affect cell growth rate. Therefore, we added different concentrations of IPTG to the medium. The *glnA* KO + AbGS showed no significant difference in cell growth across different IPTG concentrations in MOPS minimal medium containing glycerol (Fig 4D), implying that cells transferred into the medium do not require IPTG for complementation through AbGS.

Next, we conducted experiments with MOPS minimal medium containing L-glutamate and glycerol in the absence of glucose and IPTG (MOPS-GG medium). We compared *glnA* KO + pKA001 and AbGS to confirm complementation of AbGS in MOPS-GG medium. AbGS showed complementation in the *glnA* KO strain; the cells exhibited reduced growth in the absence of AbGS and complementation with AbGS statistically significantly slowed down the decay of cells (Fig 4E). We designed a strain in which AbGS and AbHeR were co-expressed in the pKA001 vector, and their expression was induced by IPTG and L-arabinose, respectively. We assumed that AbHeR without ATR cannot regulate AbGS in the absence or presence of light, whereas AbHeR with ATR regulates AbGS in the presence of light. Therefore, the growth of cells may be altered. However, the cells co-expressing AbHeR and AbGS showed decreased growth levels similar to that of *glnA* KO + pKA001 cells (Fig 4F). AbGS expression levels in the cells co-expressing AbHeR and AbGS were lower than those in the cells expressing AbGS after induction, indicating that a specific amount of AbGS is essential for complementation (S4D–S4F Fig). Although the cells co-expressing AbHeR and AbGS showed reduced growth rate, AbHeR may influence the growth of cells in the presence of light. Surprisingly, the growth of cells co-expressing AbGS and AbHeR with ATR in the presence of light was statistically significantly higher than that in the absence of light. In addition, the growth of cells expressing AbGS and AbHeR without ATR was not significantly different from that of cells expressing AbGS and AbHeR with ATR in the absence of light. That is, AbHeR could not regulate AbGS in the absence of light harvesting (Fig 4F). The low binding affinity of AbGS in the AbHeR K216Q mutant was measured using the same experimental method as that for AbHeR WT. AbHeR K216Q did not influence cell growth significantly (Fig 4G). We quantified the growth levels because it was difficult to compare the down decay of cells expressing AbGS as well as AbHeR WT or K216Q. Using exponential decay, the *t*_1/2_ of growth rate of cells containing AbHeR WT in the presence of light was 1.58 times greater than that in the absence of light (Fig 4H); however, *t*_1/2_ values of growth rates of cells containing AbHeR K216Q in presence of light were not altered when compared with those in the absence of light (Fig 4I). These *t*_1/2_ values also suggest that AbGS can be partially regulated by AbHeR in the presence of light and that AbGS does not bind well to AbHeR K216Q.

### Discussion

The heliorhodopsins studied to date do not exhibit ion-pumping activity [17,18]. The extracellular portion of heliorhodopsins comprises hydrophobic residues that restrict the movement of ions. Therefore, AbHeR does not exhibit ion-pumping activity as the space is filled with hydrophobic residues. The hydrophobic residues Tyr109, Ala113, Met116, Phe203, and Phe206 in *Thermoplasmatales* archaeon heliorhodopsin (TaHeR) corresponded to Tyr106, Ser110, Met113, Phe204, and Phe207 in AbHeR, respectively [18]. Moreover, upon comparing the hydrophobicity of heliorhodopsin and ion-pumping rhodopsin, HeR-48C12 and TaHeR exhibited hydrophobicity at amino acid positions similar to those in AbHeR. However, the regions of hydrophobicity, as analyzed by ProtScale Tool [50], differed between ion-pumping rhodopsin and heliorhodopsin significantly (S1I Fig), suggesting that the differences contribute to the lack of ion-pumping activity in AbHeR.

Nearly 4 years after the discovery of heliorhodopsin, researchers have begun proposing potential functions. For instance, Pushkarev and colleagues and Shihoya and colleagues have suggested that they function as distinct types of signaling photoreceptors due to their slow photocycle similar to that of SRs [17,18]. Additionally, enzymatic functions related to carbon fixation have been suggested for heliorhodopsin based on the adjacent gene in the genomic region. Two *nuo* loci (for NADH:ubiquinone oxidoreductase), the main entry point for electrons from NADH into the respiratory chains of most mitochondria and many bacteria, surround heliorhodopsin; however, they are not directly adjacent to it. Kovalev and colleagues suggested that a large cavity in the cytoplasmic part of heliorhodopsin might play an active site role for triangular anions, such as carbonate [19,51]. Many researchers have focused on proteins that are up- or downstream of heliorhodopsin in the operon to provide insights regarding its functions. Based on the findings of these previous studies, we postulated that the proteins encoded by both genes in the operon would interact with each other, and the large cavity in the cytoplasmic region might serve as a space to allow for conformational changes in heliorhodopsin following interaction with other proteins.

Recently, researchers have employed bioinformatics to predict function based on comparisons with genes neighboring heliorhodopsins in the genome. Bulzu and colleagues identified MORN (Membrane Occupation and Recognition Nexus, PF02493) repeat and zinc ribbon proteins (Pfam domain zinc_ribbon_4) at the N-termini of certain heliorhodopsins; these proteins were modeled to be fused to heliorhodopsins. They suggested that the proteins fused to heliorhodopsins may be related to the PPI or an indication of the role of zinc in possible downstream signaling [21]. Bulzu and colleagues and Chazan and colleagues also suggested diverse functions resulting from the relationship between heliorhodopsins and neighboring genes. The genes encode proteins related to oxidative stress, membrane transporter proteins, signaling proteins, as well as nitrogen and glucose metabolism [21,52]. We analyzed the suggested sequences of MORN repeat and zinc ribbon protein; however, we did not detect the associated sequences upstream of AbHeR. Therefore, AbHeR is not likely a fused heliorhodopsin. Not all heliorhodopsins contain a GS gene; hence, the binding of AbHeR to AbGS likely only occurs in a specific subgroup of the heliorhodopsin family, rather than in all members.

The 2 key findings of the current study are as follows: (1) AbGS is up-regulated by AbHeR in the presence of light. (2) In the absence of light, AbGS bound to AbHeR WT exhibits significantly higher activity levels than when it is not bound to AbHeR K216Q in vitro (Fig 3D–F). These results are consistent with data that the *t*_1/2_ values of glnA KO + AbGS +AbHeR WT are higher than those for glnA KO + AbGS + AbHeR K216Q in the absence of light (Fig 4H and 4I). Accordingly, the PPI between AbHeR and AbGS increases AbGS activity despite the absence of light. In addition, AbGS activity is further increased by conformational changes in AbHeR that occur in the presence of light.

According to our results, AbHeR is an AbGS-regulating heliorhodopsin; hence, other heliorhodopsins may exhibit similar functions. HeR-48C12 belonging to the GS group has sequence similarity to other members of the GS group (Fig 2A); however, it is unclear whether a single RNA polymerase can transcribe across the distance between GS and HeR-48C12, which is much larger than that for other heliorhodopsins in the GS group (Fig 1B). Moreover, amino acid sequence similarity with GS adjacent to HeR-48C12 is 36.64%, compared to 85.81% to 96.28% for GSs adjacent to other heliorhodopsins. Hence, HeR-48C12 might not bind to the adjacent GS.

The NAD^+^ synthetase belongs to the aminotransferase family involved in a variety of biochemical processes [53]. The enzyme is classified as ammonia- or glutamine-dependent and catalyzes the synthesis of NAD^+^ from nicotinic acid adenine dinucleotide (NaAD^+^) and ATP with ammonia or glutamine [53,54]. In the GS group, *Actinobacteria bacterium* IMCC26103 and other bacteria not only contain GS but also NAD^+^ synthetase genes, save for *Actinobacterium* clone fosmid 48C12 due to not analyzing the nucleotide sequence upstream of GS. Hence, GS and NAD^+^ synthetase both utilize ammonia and ATP, and both genes are under the control of a single promoter (Fig 1B). As AbGS exhibits the PPI with AbHeR, we suggest that NAD^+^ synthetase may also interact with AbHeR. Alternatively, NAD^+^ synthetase may be associated with AbGS activity in nitrogen metabolism.

Amino acid alignment of the GSs adjacent to heliorhodopsins in the GS group revealed the presence of conserved active and adenylation sites responsible for enzyme activity. In particular, Ser and Asn, previously known as the active sites, were replaced with Ala55 and Pro238 in AbGS, respectively (S6 Fig) [35]. The absence of the Ser residue (Ala55 in AbGS), which increases intersubunit stability, may facilitate its contiguous Ser54 in AbGS to substitute the intersubunit stability. In another site, the absence of Asn (Pro238 in AbGS), which coordinates the amino group of glutamate, may influence the enzyme activity, suggesting that AbGS adjacent to AbHeR exhibits low enzyme activity, excluding the GS adjacent to HeR-48C12 in the GS group owing to the absence of Ser and Asn. Interestingly, the genome of *Actinobacteria bacterium* IMCC26103 encoded 3 GSs (AbGS, QLL24912-GS, and QLL24954-GS) (S7 Fig). All residues in the active sites of QLL24954-GS were conserved. In contrast, Ser, Tyr, Asn, and Glu (Ala55, Tyr154, Pro238, and Glu302 in AbHeR) in the active sites of QLL24912-GS were replaced with Thr, Gln, Glu, and Phe, respectively. QLL24912-GS might be no catalytic activity or it might catalyze another reaction. Similarly, the absence of Asn at the Pro238 in AbGS might imply that AbGS catalyzes some other reaction. Therefore, AbGS cannot be excluded possibility as a putative GS. However, in the enzyme assay, AbGS in the presence of L-glutamate, ATP, and ammonia converted from ATP to ADP and P_i_, meaning that AbGS is most likely GS. Therefore, the binding of AbHeR to AbGS may be a strategy for overcoming the effect of AbGS with low enzyme activity.

Protein–protein docking prediction revealed that the monomer of the AbHeR dimer docked between the monomers of the AbGS dodecamer (S8 Fig). Based on the docking prediction, AbGS was raised to the phospholipid bilayer in which AbHeR was embedded. The phospholipid bilayer mediates interactions between the membrane proteins, which undergo dynamic conformational changes to perform various functions [55]. The complex of 2 membrane proteins, SR with transducer, may undergo a piston-like shift toward the cytoplasmic side by retinal isomerization [56]. Heliorhodopsins may also undergo dynamic conformational changes in the presence of light, suggesting that this change causes AbHeR to shift toward the cytoplasmic side. These reports support our docking predictions.

Previously studied GSs contain key amino acids in their active sites, including Asp, Asn, and Glu (Asp52, Pro228, Glu302 residues in AbGS), all of which were observed in the active site of AbGS, save for Asn [35]. Additionally, polar interactions between AbHeR and AbGS were measured using PyMOL. Polar interactions were observed near the key active sites, confirming that various amino acids in AbHeR and AbGS participated in the polar interaction (S8A Fig). Interestingly, important residues associated with PPIs, namely, Lys217, and Lys220 in AbHeR, are located in the key active sites and exhibit polar interactions. Moreover, Tyr212, Val218, Trp221, and Arg226 in AbHeR interact with Pro371, Gly300, Glu302, and Glu302, respectively. In addition, glutamate-binding space around the key amino acids in the active sites was sufficient to enter glutamate binding in AbGS bound to AbHeR compared to GS alone (S8B–S8E Fig). Glu302 in AbGS is located on a loop structure, termed the Glu flap, consisting of residues that guard the glutamate entrance into the active site [35]. The Glu flap in GS structures is a flexible loop that contributes glutamate entrance into the active site via switching between an open and closed conformation [57,58]. In addition, the Glu at the Glu302 position in AbGS formed hydrogen bonds with Asp and Asn in GS (Asp52 and Pro228 in AbGS) [59]. Hence, we propose that the polar interaction between Glu302 in the Glu flap of AbGS and amino acids in AbHeR, and AbHeR might cause a conformational change in the Glu flap of the glutamate entrance to the active site, in turn enhancing AbGS activity.

Furthermore, *k*_cat_ values of reported GSs were more than 2.0 [25,39–41], while the *k*_cat_ value of AbGS is lower than that of the reported GSs, exhibiting not even 1.0 turnover per second (Table 2 and S4B Fig). The absence of Asn from the key enzyme active sites (Pro228 in AbGS) may affect the enzyme catalysis of AbGS. The photocycle of heliorhodopsin is approximately 1 s faster than the *k*_cat_ value of AbGS [17]. The binding of AbHeR to the key active site of AbGS might cause a conformational change in AbGS in the absence of light. Meanwhile, in the presence of light, the conformational change might regulate the glutamate entrance through the Glu flap in a switch-type mechanism. We suggest that the binding of AbHeR to AbGS may accelerate the AbGS turnover by regulating the open and closed forms of Glu flap at the glutamate entrance in the active site.

The members of the phylum *Actinobacteria* have specific habitat preferences, including a free-living lifestyle, low-nutrient concentrations, and preference for the epilimnion layer [60]. *Actinobacteria bacterium* IMCC26103 was isolated from Lake Soyang (37° 56′ 50.6″ N, 127° 49′ 7.9″ E, South Korea) during the spring season at a depth of 50 m. The region is a freshwater environment characterized by relatively low-nutrient concentrations achieved via attenuation of physicochemical perturbations by water mixing [23]. The physicochemical characteristics, nutrient levels, and concentrations of nitrite, nitrate, and ammonium in Lake Soyang (37° 56′ 49.2″ N, 127° 48′ 57.6″ E) shift with season and depth. In particular, the concentration of nitrite in the hypolimnion layer is lower than that in the epilimnion layer during the 4 seasons. The concentration of nitrate does not differ between the hypolimnion and the epilimnion layer in any season. Ammonium is only detectable in the lake hypolimnion during spring [61]. The lake hypolimnion is an extremely low-nutrient environment, and the nutrient levels decrease gradually over time from spring to winter. Nitrite, nitrate, and ammonium are key nutrients involved in nitrogen fixation and metabolism and can be converted to ammonia, which is the substrate for GS [62]. Therefore, we hypothesized that *Actinobacteria bacterium* IMCC26103 requires nitrogen assimilation and, thus, enhanced AbGS activity. However, the active site of AbGS contains Pro238, not Asn, unlike the previously known GS enzymes. As a mechanism of enhancing AbGS activity, AbGS binds to AbHeR, thereby increasing AbGS activity in the presence of light and facilitating nitrogen assimilation.

Interestingly, the rhodopsin gene was found in *Actinobacteria bacterium* IMCC26103 and was predicted to be an H^+^-pumping rhodopsin. Moreover, genes associated with biosynthesis of retinal are essential for the formation of rhodopsin chromophore; the bacterium encodes *crtEBIY* and *blh* genes [23]. H^+^-pumping rhodopsin causes an H^+^ gradient thus altering the electrical potential difference across the membrane. Consequently, membrane-bound ATPase functions in ATP synthesis through ion gradients [63]. Habitat preferences of the bacterium may include light sources for ATP synthesis by rhodopsin via the electrical potential difference. Since the light source and concentrations of nitrite and ammonium are relatively higher in the lake epilimnion than in the lake hypolimnion, the bacterium may prefer the lake epilimnion habitat for AbGS activity with AbHeR. We propose that the bacterium can synthesize ATP by H^+^-pumping rhodopsin and can facilitate nitrogen assimilation via AbGS. Therefore, the bacterium prefers habitats exposed to light to facilitate increased growth.

In conclusion, we have demonstrated that the heliorhodopsin of *Actinobacteria bacterium* IMCC26103 regulates AbGS activity via direct binding. This interaction between AbHeR and AbGS may be critical for nitrogen assimilation in *Actinobacteria bacterium* IMCC26103 as it survives in a low-nutrient environment. Our results provide novel insights into the functioning of rhodopsin in different organisms. To the best of our knowledge, this is the first study to unveil the function of AbHeR in metabolic regulation. However, as many researchers have suggested, heliorhodopsins likely also have functions that are not associated with enzyme regulation. Therefore, further research is required to elucidate additional molecular mechanisms.

## Materials and methods

### Phylogenetic and genome analysis

Phylogenetic analysis was performed with microbial rhodopsin, reported heliorhodopsins (HeR-48C12, Actinobacterium clone fosmid 48C12; TaHeR, *Thermoplasmatales archaeon* SG8-52-1; BcHeR, *Bellilinea caldifistulae*), predicted heliorhodopsins (based on the amino acid sequence of HeR-48C12), and AbHeR. Each amino acid sequence was aligned using MUltiple Sequence Comparison by Log-Expectation (MUSCLE) [64,65]. Evolutionary analyses were inferred using the maximum likelihood method and the JTT matrix-based model. The unrooted maximum likelihood tree with the highest log likelihood (-30475.05) is shown. Initial trees were obtained by neighbor-joining algorithms to a matrix of pairwise distances estimated using a JTT model and subsequently selecting the topology with a superior log likelihood value. This analysis was used for 63 rhodopsin sequences. Evolutionary analyses were conducted using MEGA X [66]. Promoters and terminators in the genome of each eubacterium were predicted using prediction tools (phSITE, Softberry, BDGP, SAPPHIRE, iTerm-PseKNC, and ARNold) [67–72]. The gaps between genes in each eubacterium operon were predicted using the promoter and terminator prediction tools.

### Plasmid preparation

AbHeR (accession number: QLL25365)- and AbGS (accession number: QLL25366)-encoding genes containing NdeI and NotI restriction sites with a hexahistidine-tag were codon-optimized for protein expression in *E*. *coli* and chemically synthesized (Integrated DNA Technologies, United States of America). The hexahistidine-tags of AbHeR and AbGS were located at the N- and C-termini, respectively. The genes were cloned into the NdeI and NotI sites of the pET21b and pKA001 vector [46]. Each of the AbHeR mutants was prepared using site-directed mutagenesis, and the mutated gene sequences were cloned into the pET21b vector. For the complementation experiment, NcoI and SalI restriction enzyme sites were introduced into the AbHeR amplicon and introduced into pKA001-AbGS.

The AIO plasmid was designed using *E*. *coli* K-12 MG1655 and contains ampicillin and kanamycin resistance genes. The *ccdB* gene in the plasmid was removed and inserted internal ribosome entry site (IRES2) gene into sites removed the *ccdB* gene. The *IRES2* fragment was amplified by polymerase chain reaction using the 5′-AGATCTTAACCCCCCCCCTAACGTTAC-3′ (BglII) and 5′-ATGCATGTATTATCGTGTTTTTCAAAGG-3′ (NsiI) primers containing restriction enzyme sites from the MSCV-IRES-GFP vector. The *IRES2* amplicon was introduced into the All-In-One vector (Biofact, Korea), and the resultant vector was named the AIO plasmid. The GR has been studied previously [73], and the GR WT gene in the pKA001-GR WT vector was introduced into the NdeI and NotI restriction sites of the pET21b vector.

### Protein expression and purification

Rhodopsin expression and purification procedures have been previously described [8]. The AbHeR WT, AbHeR mutants, and GR WT were expressed in *E*. *coli* C43 (DE3) cultured in LB medium containing 50 μg/mL ampicillin at 37°C and 200 rpm. The cells were induced by 0.84 mM isopropyl β-D-1-thiogalactopyranoside (IPTG) and 7 μM all-*trans* retinal (ATR, Toronto Research Chemicals, Canada). After induction, the cells were harvested with a buffer S (150 mM NaCl and 50 mM Tris-HCl (pH 7.0)) and subsequently disrupted using a sonicator. Membrane fractions were isolated using ultracentrifugation (Beckman × L-90 ultracentrifuge, USA) at 100,000 *× g* and 4°C for 1 h and then solubilized with buffer S containing 1% *n*-dodecyl-β-D-maltopyranoside (DDM, Goldbio, USA) at 4°C for 16 h. The solubilized rhodopsins were prepared with Ni^2+^ NTA agarose (QIAGEN, USA) by rocking at 4°C for 4 h in the absence of light after ultracentrifugation at 4°C and 45,000 *× g* for 15 min. The protein samples were washed with buffer SD (150 mM NaCl, 50 mM Tris-HCl (pH 7.0), and 0.02% DDM) containing 25 mM imidazole and subsequently eluted with buffer SD containing 250 mM imidazole using Amicon Ultra-4 10,000 MWCO centrifugal filter units. The AbGS expression and purification procedures were slightly modified.

The *E*. *coli* BL21 (DE3) strain harboring AbGS was incubated in LB medium containing 50 μg/mL ampicillin at 37°C and 200 rpm until the optical density at 600 nm (OD_600_) reached 0.4. The cells were induced with 0.84 mM IPTG and incubated at 37°C and 200 rpm for 4 h. The cells were harvested and washed with buffer S. The harvested cells were resuspended in buffer S containing 1 mM phenylmethylsulfonyl fluoride (PMSF) and subsequently disrupted using a sonicator. The cell lysates were centrifuged at 30,000 *× g* and 4°C for 45 min. After centrifugation, the supernatants were subjected to separation using Ni^2+^ NTA agarose by rocking at 4°C for 2 h. AbGS was washed with buffer S and then eluted with buffer S containing 50 mM imidazole.

### Three-dimensional structure and protein–protein docking analysis

AbHeR and AbGS sequences were analyzed using the Swiss-Model and predicted by an available protein crystal structure HeR-48C12 (PDB code: 6su3.1.A) for AbHeR and GS (PDB code: 3qaj.1.A) for AbGS [74–76]. The predicted crystal structures were analyzed using PyMOL (The PyMOL Molecular Graphics System, Version 2.52, Schrödinger, LLC). Electrostatic potential maps of the predicted three-dimensional (3D) structures of AbHeR and AbGS were analyzed using CHARMM-GUI [77]. Protein–protein docking between the predicted crystals structure AbHeR and AbGS was conducted using ClusPro 2.0 server [78–81]. The ClusPro server performed 3 computational steps. Rigid body docking was used to sample billions of conformations using PIPER, which calculates the docked conformation energy in grids based on the fast Fourier transform (FFT) correlation approach. Root-mean-square deviation (RMSD) was generated to determine the largest clusters. Finally, the samples were refined by CHARMM minimization. The docking server resulted in 30 models of a balanced set for energy coefficients. Afterward, 1 model with a low energy value in the models was selected.

### Protein–protein interaction using ITC analysis

Previously reported methods for ITC analysis of microbial rhodopsin were followed with slight modifications [8,33]. Purified AbHeR WT, AbHeR mutants, and AbGS were completely exchanged with buffer SD using Amicon Ultra-4 10,000 MWCO centrifugal filter units and Amicon Ultra-4 30,000 MWCO centrifugal filter units, respectively. The concentrations of proteins were quantified using the Bradford assay. AbGS was continuously injected into the AbHeR at 25°C using a MicroCal ITC200 (Malvern Panalytical, United Kingdom) at the Advanced Bio-Interface Core Research Facility, Korea. ITC analysis data were evaluated using Microcal LLC ITC200 (Malvern Panalytical).

### Preparation of IMVs

Preparation of IMVs, which are ISO membrane vesicles, was performed in accordance with previously published methods with slight modifications [42,73]. After induction, the *E*. *coli* pellets containing empty vector (pET21b), AbHeR, and GR were washed with buffer P (5 mM MgSO_4_ and 50 mM potassium phosphate (pH 7.5)) instead of buffer S. The cells were homogenized to 20% (wet weight/volume) in buffer P containing 1 mM dithiothreitol (DTT) and 1 mM PMSF, and then disrupted using an EmulsiFlex-C3 high-pressure homogenizer (Avestin, Canada) with 3 passes at 10,000 psi at the Advanced Bio-Interface Core Research Facility. Unbroken cells and large debris were removed twice by centrifugation at 4°C and 39,000 *× g* for 15 min. IMVs were collected by ultracentrifugation at 4°C and 150,000 *× g* for 1.5 h. The collected IMVs were homogenized with buffer S and 100 mM NaCl, respectively, and subsequently ultracentrifuged at 4°C and 150,000 *× g* for 1.5 h. The washed IMVs were homogenized with buffer S and 100 mM NaCl, respectively, and then centrifuged at 10,000 *× g* for 5 min. The supernatants were frozen and stored in liquid nitrogen. Before use, the IMVs were thawed rapidly in a water bath at 37°C for 2 min.

### Analysis of photochemical and biophysical properties of AbHeR

The protocols for estimating p*K*a values of the counterion, preparation of RSO membrane vesicles, and the light-induced H^+^ movement assay, have been described [8]. Absorption spectra of purified AbHeR were measured using a UV-VIS spectrophotometer (Shimadzu UV-2550, Japan). The difference of absorbance form of the counterion of rhodopsin was fitted using Origin 9.0 (OriginLab, USA), and the p*K*a value of the counterion was estimated using the Henderson–Hasselbalch equation. To perform the light-induced H^+^ movement assay, membrane vesicles were prepared, resuspended, and adjusted to OD_600_ of 20 (2.0 × 10^10^ cells/mL) with 100 mM NaCl and a mixture solution (20 mM LiCl, NaCl, KCl, CsCl, and Na_2_SO_4_). The GR IMV was thawed and adjusted to OD_600_ of 15 (1.2 × 10^10^ cells/mL) with 100 mM NaCl, followed by illumination at an intensity of 100 W/m^2^ through a short-wave 500 nm cut off filter (Sigma Koki SCF-50S-44Y, Japan). The assay was performed with and without 10 μM CCCP, and the pH value was monitored using a pH meter (Horiba pH meter F-51. Japan) with a pH electrode bar (Horiba pH electrode bar 9618S-10D, Japan).

### AbGS activity with AbHeR

AbGS activity was measured by colorimetric determination of P_*i*_ concentrations using ferric sulfate and molybdate, in accordance with previous studies [36–38]. The AbHeR IMVs were adjusted to an OD_554_ of 0.330. The concentration of purified AbGS, as quantified by the Bradford assay, was adjusted to 160 μM with 50 mM imidazole-HCl (pH 7.0). First, 4 μL of each AbHeR and AbGS sample was allowed to bind at 260 rpm and 25°C for 1 h. After binding, 25 μL of an additive solution (72 mM imidazole-HCl at pH 7.0, 80 mM NH_4_Cl, 12.8 mM MnCl_2_, and 176 mM NaCl) and 5 μL of 400 mM glutamate-NaOH (pH 7.0) were added. Bound samples were incubated at 260 rpm and 25°C for 15 min. To activate enzyme activity, 2 μL of ATP solution (400 mM adenosine triphosphate (ATP) was dissolved in 1 M imidazole-HCl (pH 7.0), 150 mM NaCl, and 50 mM Tris) was added. The reaction was carried out at 37°C in the absence and presence of light (specific green laser, 532 nm) conditions at 20 μmol m^-2^ s^-1^. The reaction samples were treated with 40% trichloroacetate to be a final concentration of 20%. Treated samples were cooled at 4°C and centrifuged at 4°C and 3,500 *× g* for 30 min. Forty microliters of the supernatants containing P_*i*_ were transferred to a 96-well microplate, and 180 μL of freshly prepared reducing agent (0.8% FeSO_4_ with 0.3N H_2_SO_4_) was added. The color reaction was developed for 15 min by adding 15 μL of a freshly prepared developing solution (6.6% (NH_4_)_6_Mo7O_24_ with 7.5N H_2_SO_4_). Finally, concentrations of the developed reactants were measured at an OD_660_ using a 2300 EnSpire Multimode Plate Reader (PerkinElmer, USA). The P_*i*_ concentrations were calculated using the P_*i*_ standard curve, which was prepared using the abovementioned procedure (colorimetric determination of the P_*i*_ assay) with different concentrations of potassium phosphate buffer without AbHeR and AbGS.

### Growth rate test of cells for AbGS complementation

*E*. *coli* JW3841 (Keio collection), a *glnA*-deficient strain, was used to complement AbGS in our study [49]. The pKA001 vector contains the *lac* promoter and *araBAD* promoter with the β-dioxygenase gene. AbGS and AbHeR were regulated by P_*lac*_ and P_*araBAD*_, respectively. *E*. *coli* JW3841 was transformed with the pKA001 null vector, pKA001-AbGS, and pKA001-AbGS_AbHeR. A single colony of transformed cells was incubated in LB medium containing 50 μg/mL ampicillin and 50 μg/mL kanamycin at 37°C and 280 rpm for 16 h. The incubated cells were transferred 100:1 ratio to LB medium containing 50 μg/mL ampicillin and 50 μg/mL kanamycin at 37°C and 280 rpm until the OD_600_ reached 0.4. The cells were induced by the addition of 0.84 mM IPTG, 0.2% L-arabinose, and 7 μM ATR until the OD_600_ reached 3.0. The induced cells were washed 3 times, resuspended with buffer S, and subsequently transferred to 8 mL of MOPS minimal medium [82] (40 mM 3-morpholinopropane-1-sulfonic acid, 4 mM tricine, 10 μM F_2_SO_4_, 9.52 mM NH_4_Cl, 276 μM K_2_SO_4_, 500 nM CaCl_2_, 528 μM MgCl_2_, 50 mM NaCl, 1.32 mM K_2_HPO_4_, 3 nM (NH_4_)_6_Mo_7_O_24_, 400 nM H_3_BO_3_, 30 nM CoCl_2_, 10 nM CuSO_4_, 80 nM MnCl₂, and 10 nM ZnSO₄ (pH 7.4)) containing 0.4% carbon source (D-glucose or glycerol), 10 mM L-glutamate, 50 μg/mL ampicillin, and 50 μg/mL kanamycin to reach an OD_600_ equal to 0.1 (1.0 *×* 10^7^ cells/mL). The transferred cells were incubated at 37°C and 280 rpm in the absence and presence of light (532 nm) at 40 μmol m^-2^ s^-1^. Further OD_600_ measurements of *E*. *coli* were obtained using the UV-VIS spectrophotometer (Shimadzu UV-2550).

*E*. *coli* K-12 MG1655 was transformed with the AIO plasmid, and further experiments were performed in the same manner as described for *E*. *coli* JW3841.

### Sodium dodecyl sulfate–polyacrylamide gel electrophoresis (SDS-PAGE) and western blot

The *E*. *coli* JW3841 strain after induction of AbGS and AbHeR expression was centrifuged at 13,000 *× g* for 30 s and subsequently resuspended in Laemmli sample buffer. The samples were vigorously vortexed for 2 min and then lysed at 1,600 rpm for 20 min in a shaking incubator (Finepcr, Korea). The cell density in the buffer was equivalent to an OD_600_ value of 3.0 (3.0 × 10^8^ cells/mL). Purified GS and AbHeR were added by Laemmli sample buffer, and then boiled for 10 min and incubated at 25°C for 30 min, respectively. Prepared cell lysates and purified proteins were subjected to discontinuous SDS-PAGE that the acrylamide concentrations of stacking and separating gels are 5% and 12%, respectively. After electrophoresis, the gels were stained with Coomassie brilliant blue R-250 and developed after transferring, blocking, as well as treatment with hexahistidine-tag antibody and HRP-conjugated antibody.

### Statistical analysis

Statistical analysis was performed using the Origin 9.0 (OriginLab). The indicated values represent the mean ± standard deviation. Statistical differences between experimental groups were analyzed using 2-tailed paired *t* tests. The following *p-*values were indicated statistical significance: * *p* < 0.05, ** *p* < 0.01, *** *p* < 0.001, and **** *p* < 0.0001. The statistical analysis approaches applied in each experiment are described in the respective figure legends.

## Supporting information

S1 Fig Topological, photochemical, biophysical, and hydrophobic characteristics of AbHeR. (A) The membrane topology of AbHeR was predicted using the Philius server. The prediction reveals that AbHeR is a 7-transmembrane protein and its N-terminus is located on the cytoplasmic side. Transmembrane helix, extracellular (non-cytoplasmic) side, cytoplasmic side, and signal peptide are indicated in yellow, green, blue, and red colors, respectively. (B) Absorption spectra of AbHeR WT at different pH values. The absorption maxima at acidic, neutral, and alkaline pH were 570, 553, and 547 nm, respectively. (C and D) Differences of absorbance were calculated from the absorption spectra of AbHeR WT at different pH values. (E and F) The p*K*a values of counterion (E105) and retinal Schiff Baes were estimated using the Henderson–Hasselbalch equation. (G) AbHeR WT membrane vesicles were determined to exhibit no ion-pumping function through a light-induced proton movement assay. The mixture solution was composed of 20 mM each of LiCl, NaCl, KCl, CsCl, and Na_2_SO_4_ and was used to detect any ion pumping. The AbHeR WT membrane vesicle was measured in the absence (gray color space) and presence of light (60 to 240 s). Black and red lines indicate without and with CCCP, respectively. (H) Absorption maxima of purified AbHeR mutants at neutral pH are shown. The absorbance maxima were not significantly different compared to those of WT. (I) Hydrophobicity analyses were performed using ProtScale Tool (web.expasy.org/protscale), and amino acid scales were based on the Kyte and Doolittle method. The most frequently used scales are calculated based on hydrophobicity and hydrophilicity, and the secondary structure conformational parameter scales are calculated based on different chemical and physical properties of amino acids. Values of 0 on the y-axis are indicated by dashed gray. Heliorhodopsins, AbHeR, HeR-48C12, and *Thermoplasmatales* archaeon heliorhodopsin (TaHeR) were analyzed for similar hydrophobicity positions. Ion-pumping rhodopsins (BR, *Halobacterium salinarum* bacteriorhodopsin; GR, *Gloeobacter violaceus* rhodopsin; NaR, *Krokinobacter eikastus* Na^+^-pumping rhodopsin) were analyzed for different hydrophobicity positions among the ion-pumping rhodopsins. The heliorhodopsins contained hydrophobic residues in different positions. The underlying data of the graph can be found in S4 Data.
(TIF)

S2 Fig Amino acid alignment of microbial rhodopsin and heliorhodopsin. Multiple sequence alignments of microbial rhodopsins and heliorhodopsins were aligned using MUSCLE. Helices of AbHeR were based on the 3D structure (PDB: 6su3.1.A), which was predicted using the Swiss-Model. Counterions of bacteriorhodopsin and heliorhodopsin are indicated by black and red triangles, respectively. Retinal-binding pocket, retinal covalent linkage, and positions of mutation for AbHeR are indicated by black star, red star, and black plus mark, respectively. Columns indicating membrane topology are shown: BR (pink), AbHeR (sky blue), HeR-48C12 (purple), intracellular loop (green), and extracellular loop (green). Negatively and positively charged residues near intracellular loops of heliorhodopsin are indicated by red and blue, respectively. (A) Sequence alignment of AbHeR with microbial rhodopsin. ChR1, *Volvox carteri f*. *nagariensis* ChR1 (300 amino acids of full sequences); ACR1, *Guillardia theta* anion channelrhodopsin 1; ASR, *Anabaena* sp. PCC7120 sensory rhodopsin; BR, *Halobacterium salinarum* bacteriorhodopsin; GR, *Gloeobacter violaceus* rhodopsin; NaR, *Krokinobacter eikastus* Na^+^-pumping rhodopsin; NmCIR, *Nonlabens marinus* Cl^-^-pumping rhodopsin. (B) Alignment of AbHeR among the reported and predicted heliorhodopsins. Reported heliorhodopsins, HeR-48C12, Actinobacterium clone fosmid 48C12; TaHeR, *Thermoplasmatales archaeon* SG8-52-1; BcHeR, *Bellilinea caldifistulae*. Predicted heliorhodopsins, 29HeR, *Humibacillus* sp. DSM 29435 HeR; 137HeR, *Nocardioides terrigena* HeR; 208HeR, *Williamsia herbipolensis* HeR; 265HeR, *Trichococcus flocculiformis* HeR; 293HeR, *Streptomyces pini* HeR.
(TIF)

S3 Fig Predicted 3D structures and electrostatic potential maps of AbHeR and AbGS. (A) Side and (B) bottom views of AbHeR (PDB code: 6su3.1.A). (C) Side and (D) bottom views of AbGS (PDB code: 3qaj.1.A). The dimer and dodecamer formed from 2 face-to-face hexameric rings of subunits, respectively, are shown. Negative and positive values in the bottom bar indicate negatively and positively charged fields, respectively. (E) Charged fields in AbGS were difficult to distinguish. (F) AbHeR was viewed from various angles; it exhibited a dominant positively charged field on the cytoplasmic side. Each of the charged residues is indicated. Positions of cytoplasmic and extracellular sides are indicated near structures with white arrows.
(TIF)

S4 Fig Determination of AbGS activity and expression level of AbGS and AbHeR. AbGS activity is determined by P_*i*_ via biosynthetic reaction that GS catalyzes from L-glutamate with ammonia, ATP, and metal ion, to L-glutamine, ADP, P_*i*_, and proton. The biosynthetic reaction was carried out at 37°C and performed by colorimetric determination of P_*i*_ using ferric sulfate and molybdate. (A) P_*i*_ standard curve with different P_*i*_ concentrations was fitted in a quadratic equation; the equation and R-squared values are indicated. A biosynthesis assay was performed and the enzyme activity was calculated using the P_*i*_ standard curve. The initial rates (V_0_) of biosynthesis via GS reactions are expressed as μM of P_*i*_ produced per minute. The enzyme kinetic parameters were calculated using the Michaelis–Menten equation (B) and Lineweaver–Burk plot (C). (A–C) These assays were performed in an independent experimental group (*n* = 3). Data are the mean ± standard deviation. (D and E) SDS-PAGE and western blot of cell lysates and purified proteins. Cell lysates of *E*. *coli* JW3841 containing AbGS or AbGS as well as AbHeR, with and without all-*trans* retinal. Purified GS and AbHeR were used as control. Black and red arrows indicate AbGS (predicted size of AbGS monomer with hexahistidine-tag: 49.1 kDa) and AbHeR monomer bands (predicted size of AbHeR monomer with hexahistidine-tag: 28.7 kDa), respectively. AbGS was successfully expressed in both single expression and co-expressing strains; however, it exhibited lower expression in co-expressing cells. (F) *E*. *coli* JW3841 pellets containing AbHeR and AbGS are observed in pink. The underlying data of the graph can be found in S5 Data.
(TIF)

S5 Fig Photochemical and biochemical properties, as well as thermal and light stability of prepared IMV containing rhodopsins. (A) The absorption spectra of AbHeR and empty vector IMV were measured. (B) H^+^-pumping in *Gloeobacter* rhodopsin (GR) WT IMV was measured using light-induced proton movement assay. The AbHeR WT IMVs were analyzed in the absence (gray color space) and presence of light (60 to 240 s). Black and red lines indicate reactions with and without CCCP, respectively. (C) AbHeR WT IMV was incubated in light (532 nm) at 55 μmol m^-2^s^-1^ and 37°C. This test was performed in an independent experimental group (*n* = 3). Data are the mean ± standard deviation. The underlying data of the graph can be found in S6 Data.
(TIF)

S6 Fig Amino acid alignment of GS with neighboring heliorhodopsins in the GS group. The GS enzymes in the GS group are highly conserved based on their amino acid sequences; the active sites, key active sites, and adenylation site of GS are indicated in black, red, and blue circles, respectively. The GS-encoding genes and neighboring heliorhodopsin and reported heliorhodopsin were aligned. Predicted heliorhodopsins in GS group, 2HeR, Candidatus *Planktophila limnetica* HeR; 6HeR, *Actinobacteria bacterium* IMCC25003 HeR; 21HeR, Candidatus *Planktophila versatilis* HeR; 26HeR, Candidatus *Planktophila lacus* HeR.
(TIF)

S7 Fig Amino acid alignment of AbGS with GSs in genome of *Actinobacteria bacterium* IMCC21603. The GS enzymes in the genome of *Actinobacteria bacterium* IMCC21603 are conserved based on their amino acid sequences; the active sites, key active sites, and adenylation site of GS are indicated in black, red, and blue circles, respectively. The predicted GSs (Accession number: QLL24912 and QLL24954) were labeled to under accession number-GS.
(TIF)

S8 Fig Protein–protein docking to predict the 3D protein structure of AbHeR and AbGS. AbHeR and AbGS are indicated as transparent cyan and red helices, respectively. The distances between the hydrogen bonds of interacting amino acids of AbHeR and AbGS were calculated by polar interaction tool in PyMOL and indicated using a yellow dotted line. The key active sites of AbGS are present in the 2 AbGS monomers of dodecamer, and the positions of the key active sites of the 2 different monomers in AbGS are indicated in yellow and white text. The positions of amino acids in AbHeR are indicated in blue text. (A, D, and E) Docking parts of key active sites in AbGS and AbHeR. (B and C) GS 3D structure (PDB: 6su3.1.A) in a position with bound Glu. (D and E) The positions of the docking prediction are indicated with white dotted circles. (B and D) Above and (C and E) top views of the positions are shown.
(TIF)

S1 Raw Images Original images showing SDS–PAGE and western blot in S4D and S4E Fig. (PDF)

S1 Data Underlying numerical data of protein–protein interaction using isothermal titration calorimetry analysis. (XLSX)

S2 Data Underlying numerical data of GS activity with AbHeR using biosynthesis assay in the absence and presence of light. (XLSX)

S3 Data Underlying numerical data of growth rate test of *E*. *coli*. (XLSX)

S4 Data Underlying numerical data of characterization of AbHeR. (XLSX)

S5 Data Underlying numerical data of inorganic phosphate standard curve and GS activity using biosynthesis assay. (XLSX)

S6 Data Underlying numerical data of properties of IMVs containing rhodopsins. (XLSX)

## Abbreviations

AbHeR: *Actinobacteria bacterium* IMCC26103 heliorhodopsin
ADP: adenosine diphosphate
AIO: all-in-one
ATP: adenosine triphosphate
BR: bacteriorhodopsin
CP: cytoplasmic
DTT: dithiothreitol
EC: extracellular
FFT: fast Fourier transform
GS: glutamine synthetase
ICL: intracellular loop
IMV: inverted membrane vesicle
ITC: isothermal titration calorimetry
KO: knockout
LB: Luria–Bertani
MUSCLE: MUltiple Sequence Comparison by Log-Expectation
PMSF: phenylmethylsulfonyl fluoride
PPI: protein–protein interaction
RMSD: root-mean-square deviation
RSO: right-side-out
SR: sensory rhodopsin
WT: wild type
