# Supplementary figures and images for: Heliorhodopsin binds and regulates glutamine synthetase activity
Source: PLoS Biol. 2022 Oct 3;20(10):e3001817. doi: 10.1371/journal.pbio.3001817 (PMC9529153; doi:10.1371/journal.pbio.3001817)

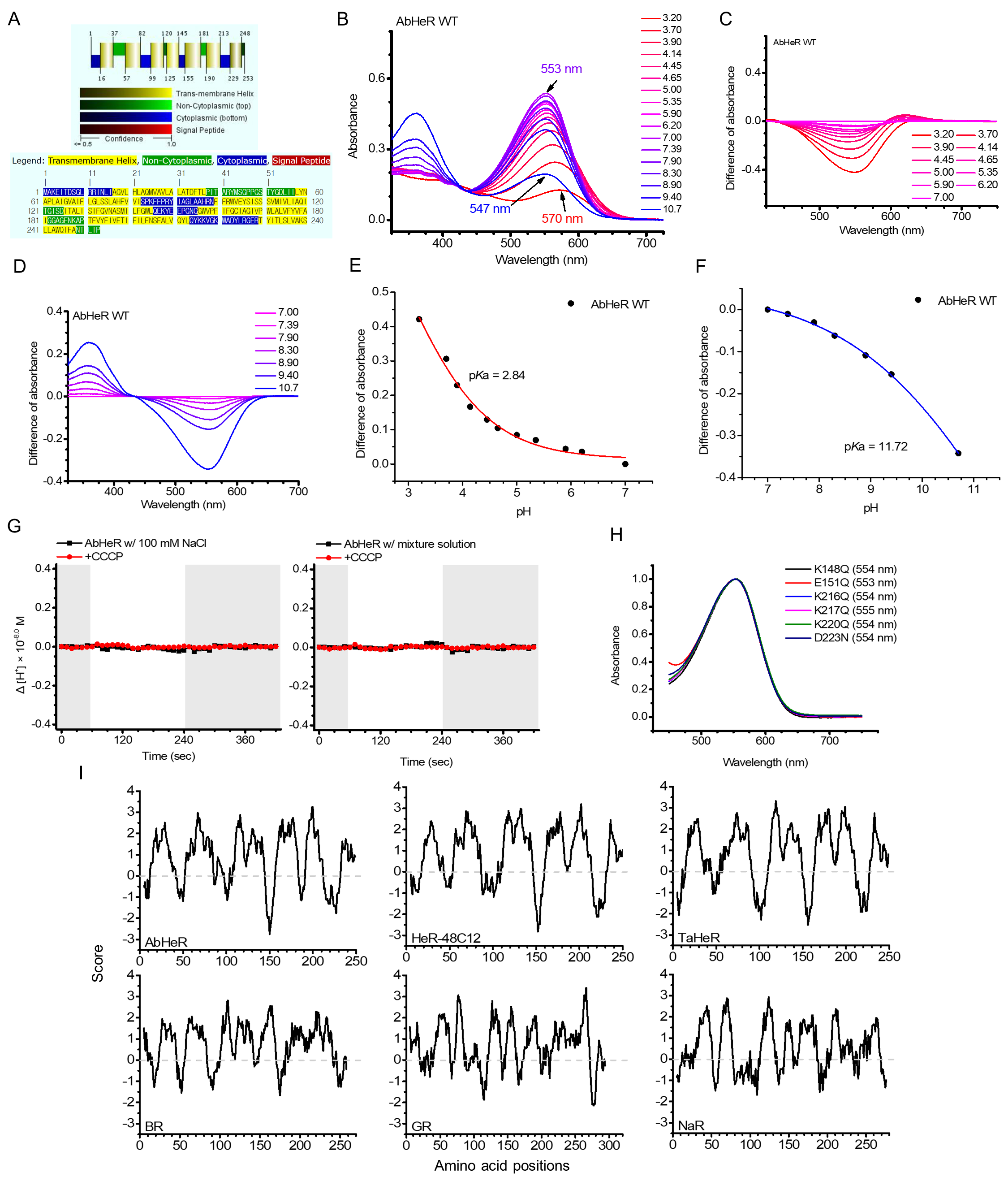

Supplement: S1 Fig — (A) The membrane topology of AbHeR was predicted using the Philius server. The prediction reveals that AbHeR is a 7-transmembrane protein and its N-terminus is located on the cytoplasmic side. Transmembrane helix, extracellular (non-cytoplasmic) side, cytoplasmic side, and signal peptide are indicated in yellow, green, blue, and red colors, respectively. (B) Absorption spectra of AbHeR WT at different pH values. The absorption maxima at acidic, neutral, and alkaline pH were 570, 553, and 547 nm, respectively. (C and D) Differences of absorbance were calculated from the absorption spectra of AbHeR WT at different pH values. (E and F) The pKa values of counterion (E105) and retinal Schiff Baes were estimated using the Henderson–Hasselbalch equation. (G) AbHeR WT membrane vesicles were determined to exhibit no ion-pumping function through a light-induced proton movement assay. The mixture solution was composed of 20 mM each of LiCl, NaCl, KCl, CsCl, and Na2SO4 and was used to detect any ion pumping. The AbHeR WT membrane vesicle was measured in the absence (gray color space) and presence of light (60 to 240 s). Black and red lines indicate without and with CCCP, respectively. (H) Absorption maxima of purified AbHeR mutants at neutral pH are shown. The absorbance maxima were not significantly different compared to those of WT. (I) Hydrophobicity analyses were performed using ProtScale Tool (web.expasy.org/protscale), and amino acid scales were based on the Kyte and Doolittle method. The most frequently used scales are calculated based on hydrophobicity and hydrophilicity, and the secondary structure conformational parameter scales are calculated based on different chemical and physical properties of amino acids. Values of 0 on the y-axis are indicated by dashed gray. Heliorhodopsins, AbHeR, HeR-48C12, and Thermoplasmatales archaeon heliorhodopsin (TaHeR) were analyzed for similar hydrophobicity positions. Ion-pumping rhodopsins (BR, Halobacterium salinarum bacteriorhodo [file pbio.3001817.s001.TIF]

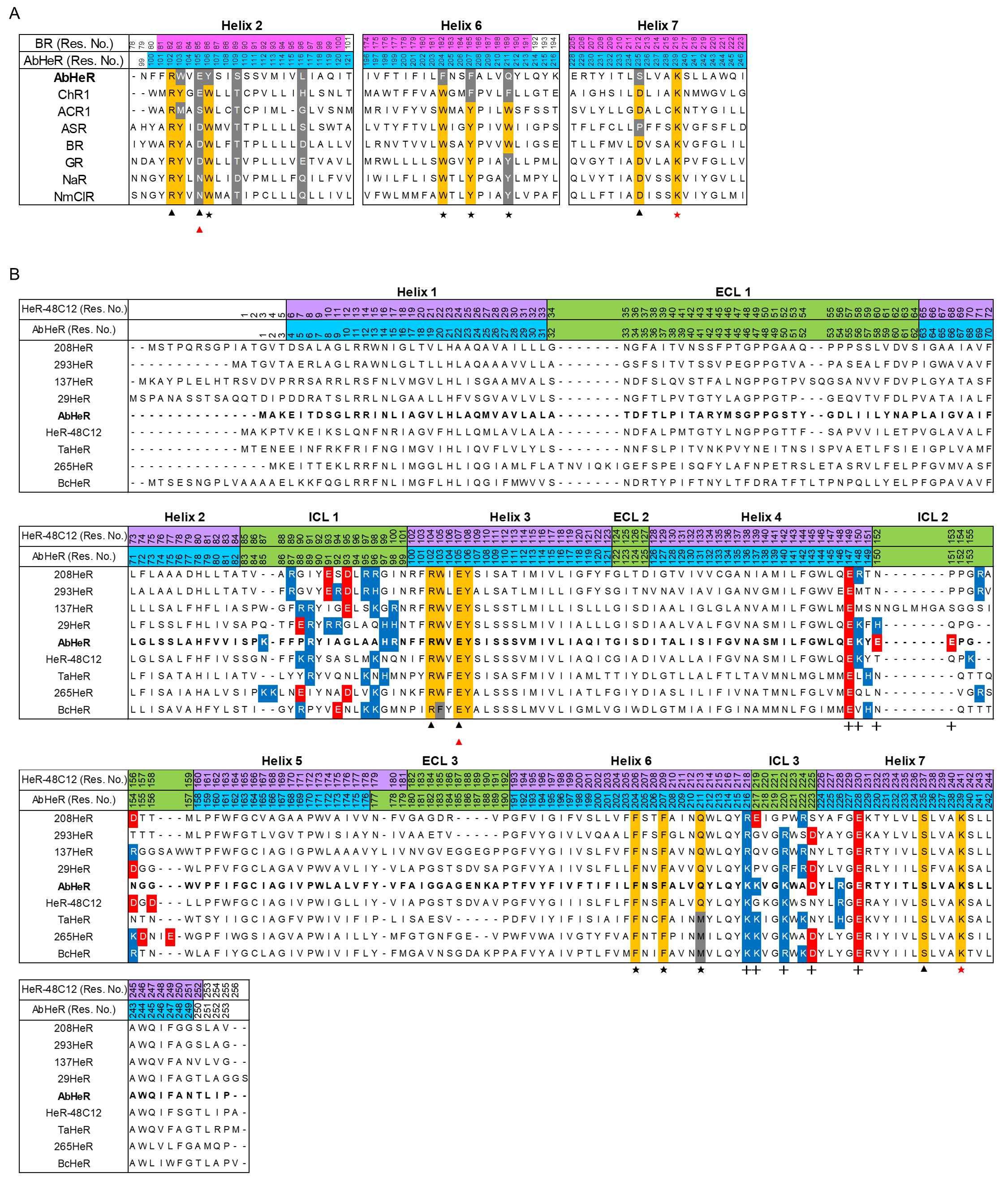

Supplement: S2 Fig — Multiple sequence alignments of microbial rhodopsins and heliorhodopsins were aligned using MUSCLE. Helices of AbHeR were based on the 3D structure (PDB: 6su3.1.A), which was predicted using the Swiss-Model. Counterions of bacteriorhodopsin and heliorhodopsin are indicated by black and red triangles, respectively. Retinal-binding pocket, retinal covalent linkage, and positions of mutation for AbHeR are indicated by black star, red star, and black plus mark, respectively. Columns indicating membrane topology are shown: BR (pink), AbHeR (sky blue), HeR-48C12 (purple), intracellular loop (green), and extracellular loop (green). Negatively and positively charged residues near intracellular loops of heliorhodopsin are indicated by red and blue, respectively. (A) Sequence alignment of AbHeR with microbial rhodopsin. ChR1, Volvox carteri f. nagariensis ChR1 (300 amino acids of full sequences); ACR1, Guillardia theta anion channelrhodopsin 1; ASR, Anabaena sp. PCC7120 sensory rhodopsin; BR, Halobacterium salinarum bacteriorhodopsin; GR, Gloeobacter violaceus rhodopsin; NaR, Krokinobacter eikastus Na+-pumping rhodopsin; NmCIR, Nonlabens marinus Cl--pumping rhodopsin. (B) Alignment of AbHeR among the reported and predicted heliorhodopsins. Reported heliorhodopsins, HeR-48C12, Actinobacterium clone fosmid 48C12; TaHeR, Thermoplasmatales archaeon SG8-52-1; BcHeR, Bellilinea caldifistulae. Predicted heliorhodopsins, 29HeR, Humibacillus sp. DSM 29435 HeR; 137HeR, Nocardioides terrigena HeR; 208HeR, Williamsia herbipolensis HeR; 265HeR, Trichococcus flocculiformis HeR; 293HeR, Streptomyces pini HeR. (TIF) [file pbio.3001817.s002.tif]

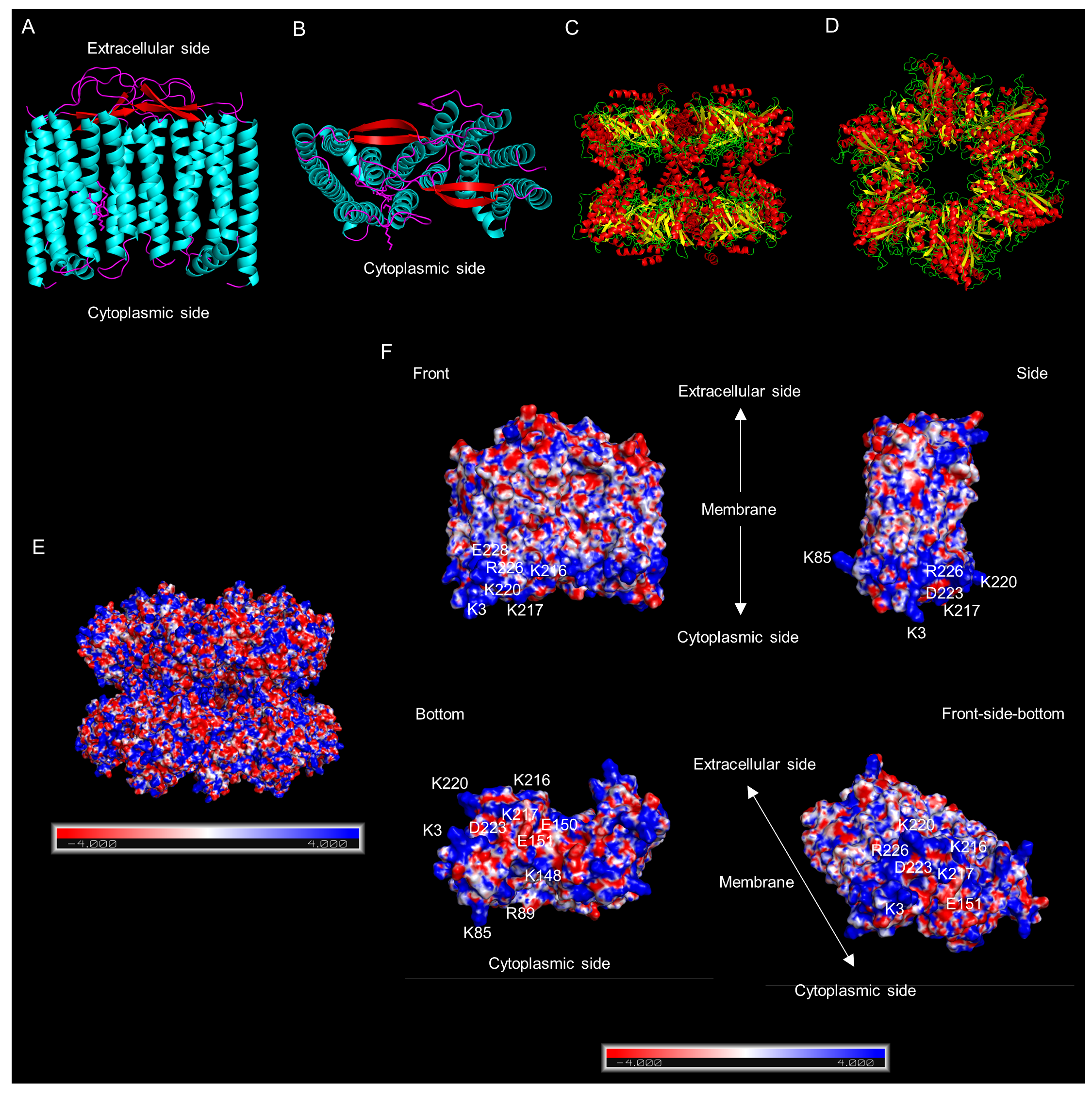

Supplement: S3 Fig — (A) Side and (B) bottom views of AbHeR (PDB code: 6su3.1.A). (C) Side and (D) bottom views of AbGS (PDB code: 3qaj.1.A). The dimer and dodecamer formed from 2 face-to-face hexameric rings of subunits, respectively, are shown. Negative and positive values in the bottom bar indicate negatively and positively charged fields, respectively. (E) Charged fields in AbGS were difficult to distinguish. (F) AbHeR was viewed from various angles; it exhibited a dominant positively charged field on the cytoplasmic side. Each of the charged residues is indicated. Positions of cytoplasmic and extracellular sides are indicated near structures with white arrows. (TIF) [file pbio.3001817.s003.TIF]

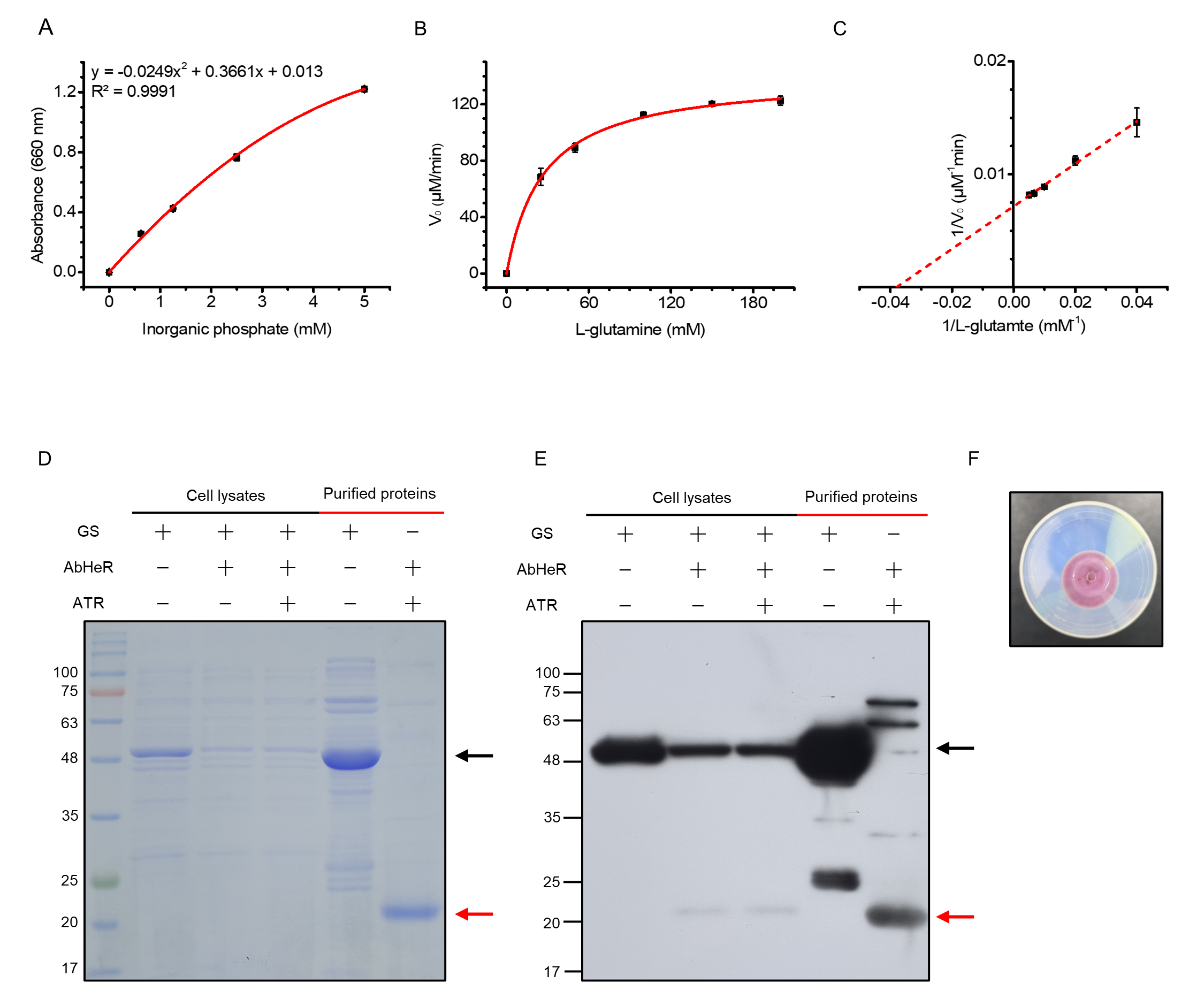

Supplement: S4 Fig — AbGS activity is determined by Pi via biosynthetic reaction that GS catalyzes from L-glutamate with ammonia, ATP, and metal ion, to L-glutamine, ADP, Pi, and proton. The biosynthetic reaction was carried out at 37°C and performed by colorimetric determination of Pi using ferric sulfate and molybdate. (A) Pi standard curve with different Pi concentrations was fitted in a quadratic equation; the equation and R-squared values are indicated. A biosynthesis assay was performed and the enzyme activity was calculated using the Pi standard curve. The initial rates (V0) of biosynthesis via GS reactions are expressed as μM of Pi produced per minute. The enzyme kinetic parameters were calculated using the Michaelis–Menten equation (B) and Lineweaver–Burk plot (C). (A–C) These assays were performed in an independent experimental group (n = 3). Data are the mean ± standard deviation. (D and E) SDS-PAGE and western blot of cell lysates and purified proteins. Cell lysates of E. coli JW3841 containing AbGS or AbGS as well as AbHeR, with and without all-trans retinal. Purified GS and AbHeR were used as control. Black and red arrows indicate AbGS (predicted size of AbGS monomer with hexahistidine-tag: 49.1 kDa) and AbHeR monomer bands (predicted size of AbHeR monomer with hexahistidine-tag: 28.7 kDa), respectively. AbGS was successfully expressed in both single expression and co-expressing strains; however, it exhibited lower expression in co-expressing cells. (F) E. coli JW3841 pellets containing AbHeR and AbGS are observed in pink. The underlying data of the graph can be found in S5 Data. (TIF) [file pbio.3001817.s004.TIF]

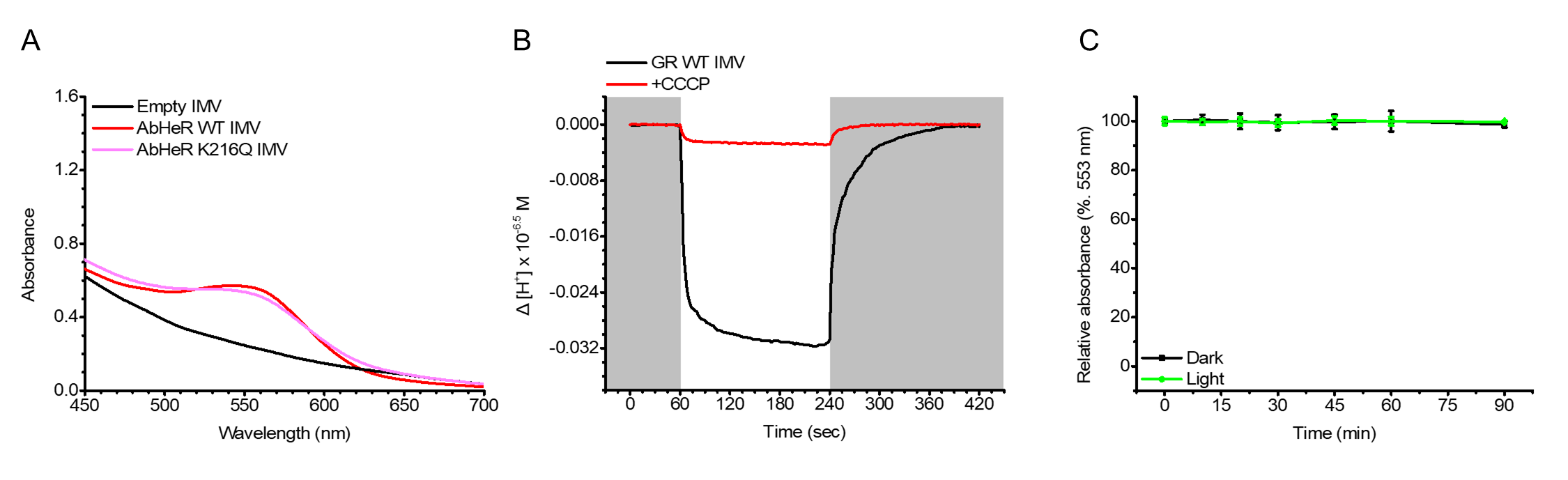

Supplement: S5 Fig — (A) The absorption spectra of AbHeR and empty vector IMV were measured. (B) H+-pumping in Gloeobacter rhodopsin (GR) WT IMV was measured using light-induced proton movement assay. The AbHeR WT IMVs were analyzed in the absence (gray color space) and presence of light (60 to 240 s). Black and red lines indicate reactions with and without CCCP, respectively. (C) AbHeR WT IMV was incubated in light (532 nm) at 55 μmol m-2s-1 and 37°C. This test was performed in an independent experimental group (n = 3). Data are the mean ± standard deviation. The underlying data of the graph can be found in S6 Data. (TIF) [file pbio.3001817.s005.TIF]

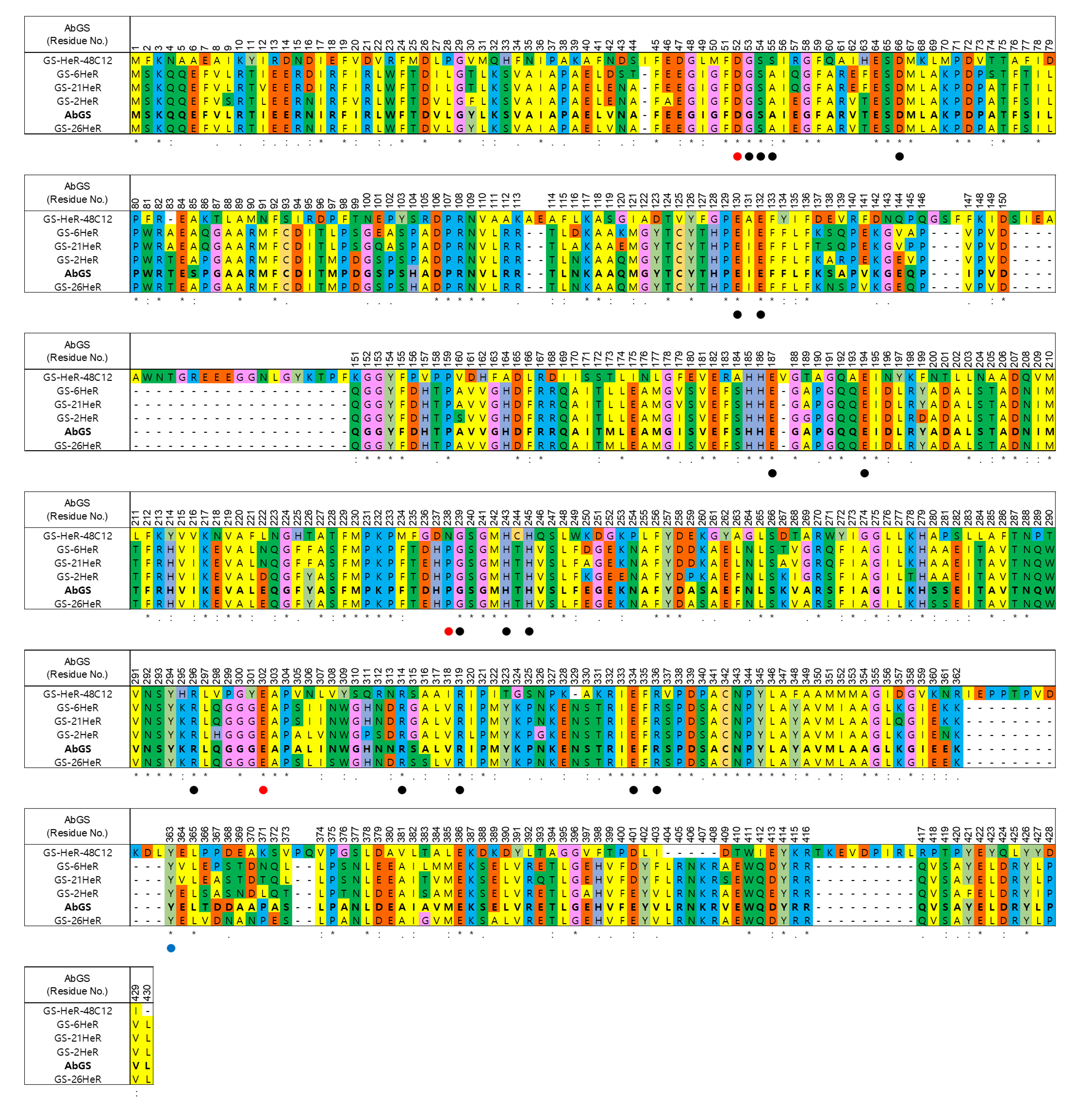

Supplement: S6 Fig — The GS enzymes in the GS group are highly conserved based on their amino acid sequences; the active sites, key active sites, and adenylation site of GS are indicated in black, red, and blue circles, respectively. The GS-encoding genes and neighboring heliorhodopsin and reported heliorhodopsin were aligned. Predicted heliorhodopsins in GS group, 2HeR, Candidatus Planktophila limnetica HeR; 6HeR, Actinobacteria bacterium IMCC25003 HeR; 21HeR, Candidatus Planktophila versatilis HeR; 26HeR, Candidatus Planktophila lacus HeR. (TIF) [file pbio.3001817.s006.TIF]

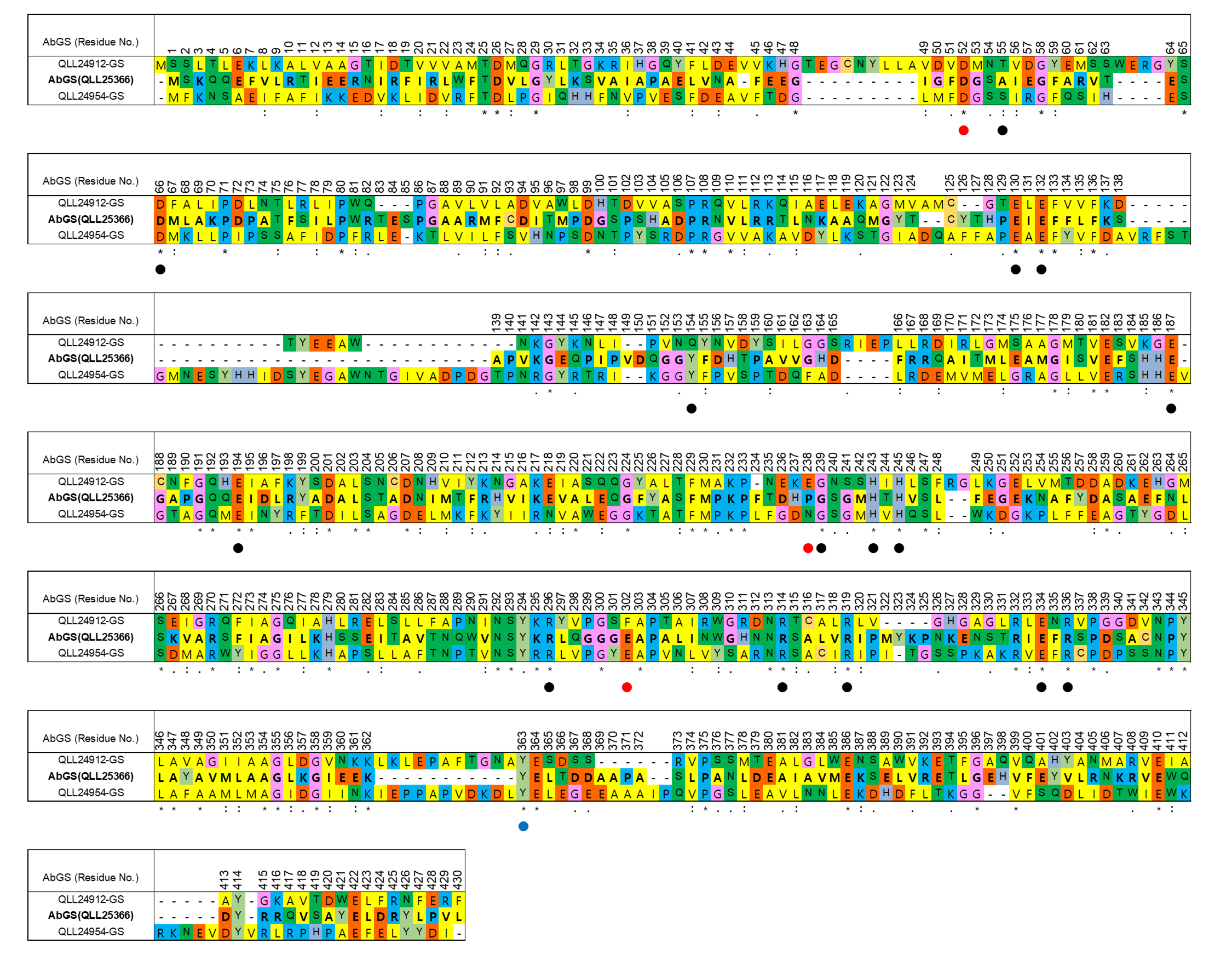

Supplement: S7 Fig — The GS enzymes in the genome of Actinobacteria bacterium IMCC21603 are conserved based on their amino acid sequences; the active sites, key active sites, and adenylation site of GS are indicated in black, red, and blue circles, respectively. The predicted GSs (Accession number: QLL24912 and QLL24954) were labeled to under accession number-GS. (TIF) [file pbio.3001817.s007.TIF]

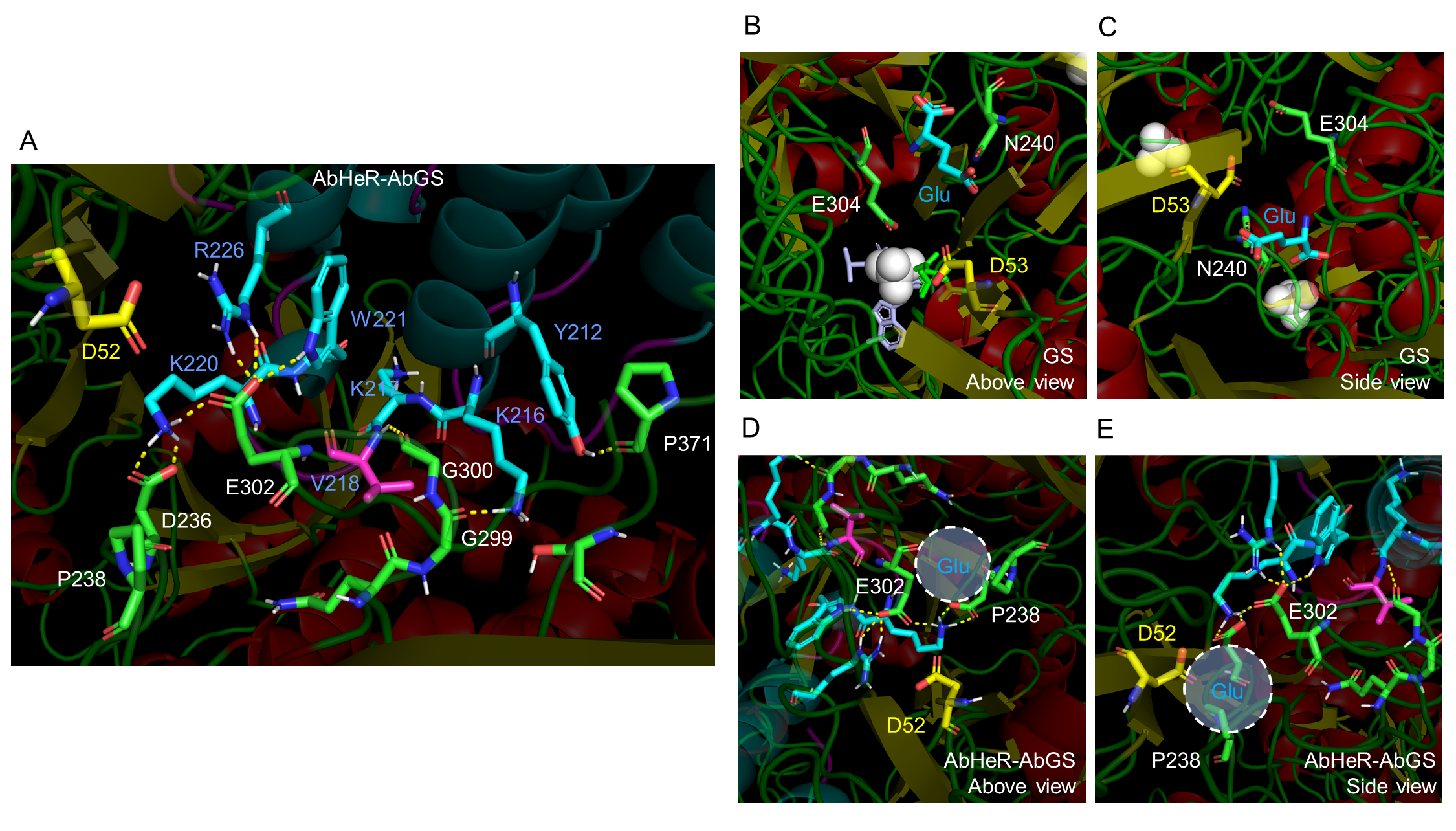

Supplement: S8 Fig — AbHeR and AbGS are indicated as transparent cyan and red helices, respectively. The distances between the hydrogen bonds of interacting amino acids of AbHeR and AbGS were calculated by polar interaction tool in PyMOL and indicated using a yellow dotted line. The key active sites of AbGS are present in the 2 AbGS monomers of dodecamer, and the positions of the key active sites of the 2 different monomers in AbGS are indicated in yellow and white text. The positions of amino acids in AbHeR are indicated in blue text. (A, D, and E) Docking parts of key active sites in AbGS and AbHeR. (B and C) GS 3D structure (PDB: 6su3.1.A) in a position with bound Glu. (D and E) The positions of the docking prediction are indicated with white dotted circles. (B and D) Above and (C and E) top views of the positions are shown. (TIF) [file pbio.3001817.s008.TIF]

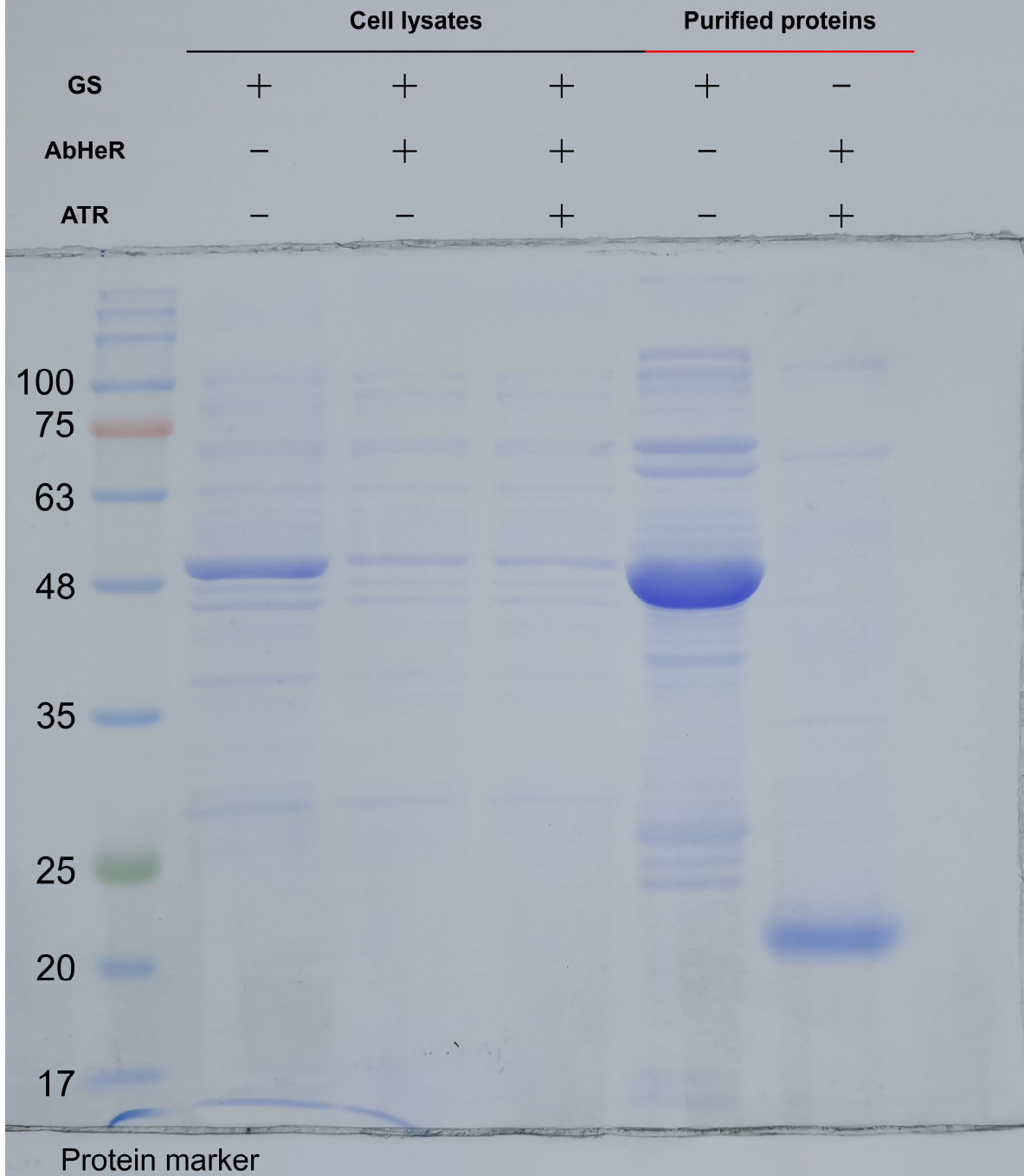

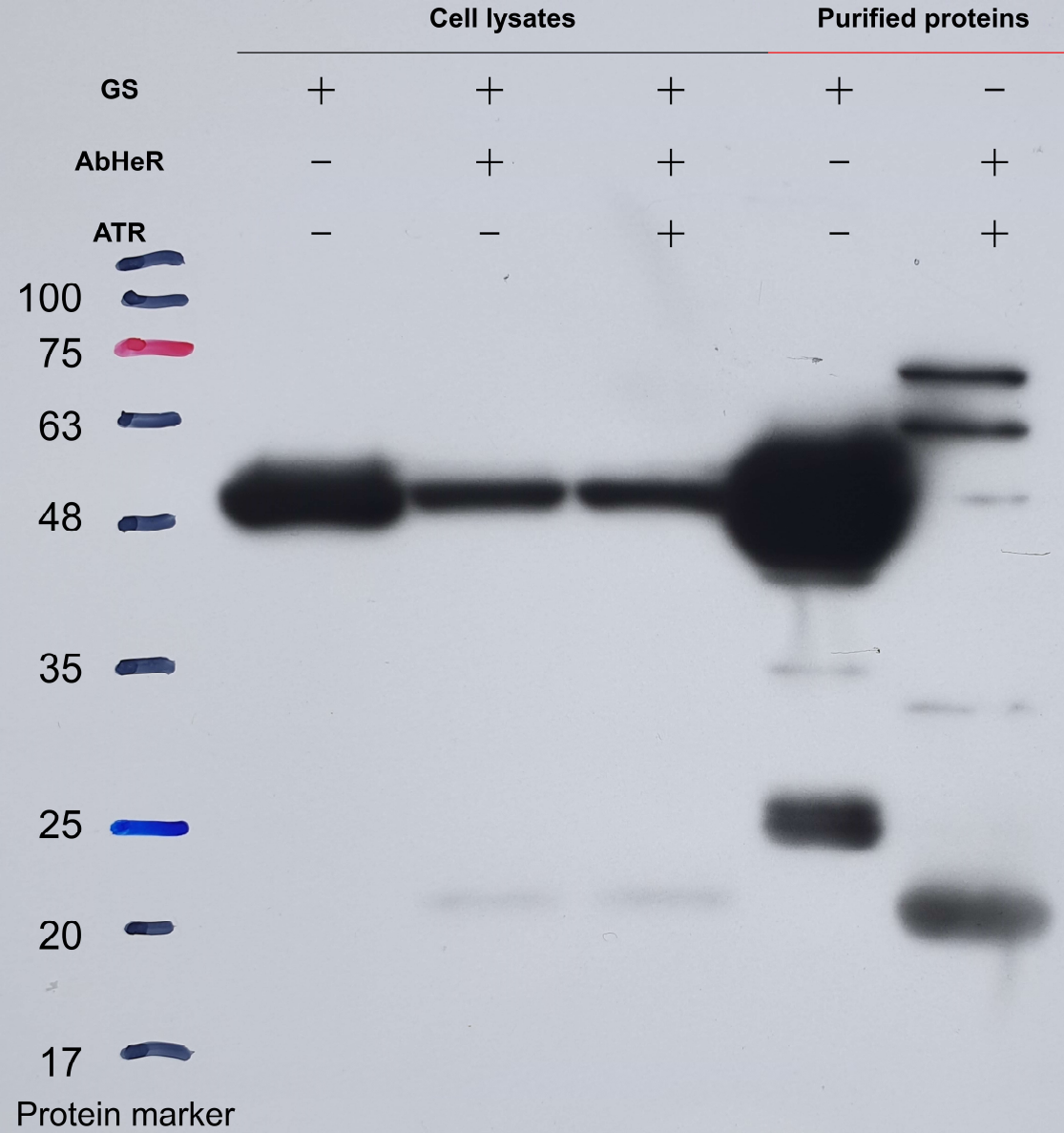

Supplement: S1 Raw Images — (PDF) [file pbio.3001817.s009.pdf]
